# Core enhancers of the 3′RR optimize *IgH* nuclear position and loop conformation for successful oriented class switch recombination

**DOI:** 10.1093/nar/gkae867

**Published:** 2024-10-16

**Authors:** Charlotte Bruzeau, Ophélie Martin, Justine Pollet, Morgane Thomas, Zhaoqing Ba, David Roulois, Eric Pinaud, Sandrine Le Noir

**Affiliations:** UMR CNRS7276, Inserm1262, Université de Limoges: Contrôle de la Réponse Immune B et des Lymphoproliférations, Team 2, B-NATION: B Cell Nuclear Architecture, Immunoglobulin Genes and Oncogenes, 2, rue du Dr. Marcland, 87025 Limoges, France; UMR CNRS7276, Inserm1262, Université de Limoges: Contrôle de la Réponse Immune B et des Lymphoproliférations, Team 2, B-NATION: B Cell Nuclear Architecture, Immunoglobulin Genes and Oncogenes, 2, rue du Dr. Marcland, 87025 Limoges, France; UMR CNRS7276, Inserm1262, Université de Limoges: Contrôle de la Réponse Immune B et des Lymphoproliférations, Team 2, B-NATION: B Cell Nuclear Architecture, Immunoglobulin Genes and Oncogenes, 2, rue du Dr. Marcland, 87025 Limoges, France; UMR CNRS7276, Inserm1262, Université de Limoges: Contrôle de la Réponse Immune B et des Lymphoproliférations, Team 2, B-NATION: B Cell Nuclear Architecture, Immunoglobulin Genes and Oncogenes, 2, rue du Dr. Marcland, 87025 Limoges, France; National Institute of Biological Sciences, 37WH+XG9, Changping District, Beijing 102206, China; Honeycomb team, Equipe Labellisée par la Ligue Nationale contre le Cancer, UMR 1236, Université de Rennes, INSERM, Établissement Français du Sang Bretagne, 2 avenue du professeur Léon Bernard, F-35043, Rennes, France; UMR CNRS7276, Inserm1262, Université de Limoges: Contrôle de la Réponse Immune B et des Lymphoproliférations, Team 2, B-NATION: B Cell Nuclear Architecture, Immunoglobulin Genes and Oncogenes, 2, rue du Dr. Marcland, 87025 Limoges, France; UMR CNRS7276, Inserm1262, Université de Limoges: Contrôle de la Réponse Immune B et des Lymphoproliférations, Team 2, B-NATION: B Cell Nuclear Architecture, Immunoglobulin Genes and Oncogenes, 2, rue du Dr. Marcland, 87025 Limoges, France

## Abstract

In B lymphocytes, class switch recombination (CSR) is an essential process that adapts immunoglobulin (Ig) subtypes to antigen response. Taking place within the Ig heavy chain (*IgH*) locus, CSR needs controlled transcription of targeted regions governed by the *IgH 3′ regulatory region* (*3′RR*). This super-enhancer is composed of four core enhancers surrounded by inverted repeated sequences, forming a quasi-palindrome. In addition to transcription, nuclear organization appears to be an important level in CSR regulation. While it is now established that chromatin loop extrusion takes place within *IgH* locus to facilitate CSR by bringing the donor and acceptor switch regions closer together, the underlying mechanism that triggers CSR loop formation remains partially understood. Here, by combining DNA 3D fluorescence *in situ*hybridization with various high-throughput approaches, we deciphered critical functions for the *3′RR* core enhancer element in nuclear addressing, accessibility and chromatin looping of the *IgH* locus. We conclude that the *3′RR* core enhancers are necessary and sufficient to pre-organize the position and conformation of *IgH* loci in resting B-cell nuclei to enable the deletional recombination events required for productive successful CSR in activated B-cell nuclei.

## Graphical Abstract

**Graphical Abstract:**
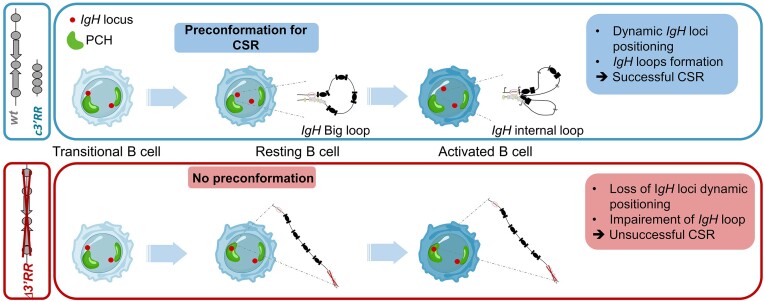

## Introduction

**Figure 1.**
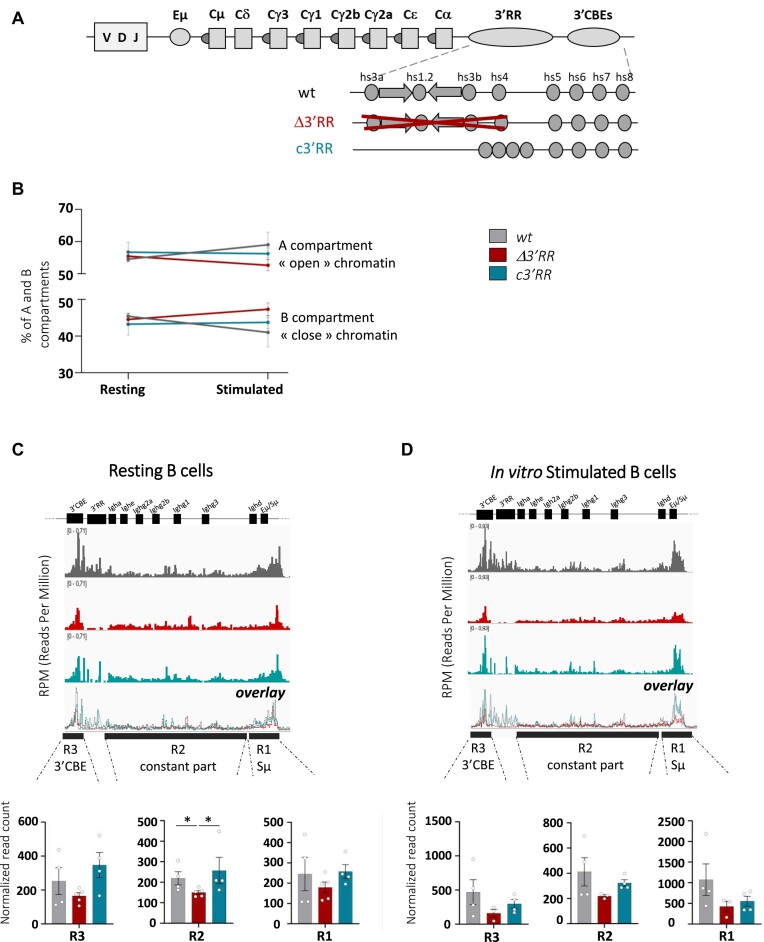
*3′RR* core enhancers maintain *IgH* loci in an active compartment in mature resting B cells. (**A**) Scheme of the murine *IgH* locus (not to scale) including the components of the *3′RR* and the mouse models used in this study carrying Δ*3′RR* (6,35) and *c’3RR* (7) deletions. *IgH* constant genes are represented by squares, core enhancers by gray circles, palindromic structure by arrows. (**B**) A and B compartment proportions determined by Compartmap (33) in resting and stimulated B cells from *wt* and mutants (**C**) Top: *IgH* locus accessibility assayed by ATAC-seq (assay for transposase-accessible chromatin with sequencing) in splenic resting B cells from *wt* (*n* = 4 mice), *3′RR* (*n* = 5 mice) and *c’3RR* mice (*n* = 4 mice) independent mice. Bedgraphs are normalized to reads per million and visualized with IGV tool. Middle: *IgH* loci were divided into three regions and the normalized read coverage was counted in each region as indicated in each bargraph. (**D**) As in panel (**B**) for *in vitro* LPS activated B cells from *wt* (*n* = 4 independent mice), *Δ3′RR* (*n* = 3 mouse) and *c3′RR* (*n* = 4 independent mice). Error bars represent SEM (standard error of mean). *P*-value was determined by a two-tailed Mann–Whitney test; only significant differences are indicated (*P< 0.05).

Late stages of B-cell development are characterized by the remodeling of immunoglobulin genes to produce highly specific antibodies. Somatic hypermutation (SHM) and class switch recombination (CSR) are the two main immunoglobulin heavy chain (*IgH*) locus modifications that both require transcription of target sequences and cytidine deamination by the activation-induced cytidine deaminase (AID) enzyme (1). It has been extensively documented in the literature that these events are under the transcriptional control of the super-enhancer located at *IgH* locus 3′ end: the *IgH 3′ regulatory region* (*3′RR*) (2). In mice, this region is composed of four core enhancers, (*hs3a*, *hs1.2*, *hs3b* and *hs4*), which are surrounded by inverted repeated sequences (IRIS) centered on *hs1.2* enhancer and delimited by *hs3a* and *hs3b* (3). This singular configuration globally forms one of the largest quasi-palindrome in the genome (3) (Figure 1A). *IgH* locus forms a topologically associated domain (TAD) as demonstrated by chromosome conformation capture (3C)-based studies (4). The 3′ end of the locus is characterized by an insulator region composed of 10 CTCF (CCCTC-binding factor) binding sites named 3′ CTCF binding elements (*3′CBE*), which delimits the 3′ *IgH* TAD border (5). Several mouse models carrying partial or total deletions of the *3′RR* region allow to decipher the exact role of both core enhancers and quasi-palindromic structure on SHM, CSR, transcription and immunoglobulin production during B-cell development (3). Regarding CSR, the *3′RR* entire deletion (Δ*3′RR* model) leads to a 2–5-fold decrease of *IgH* germline transcription (GLT) at *S* regions (6) and consequently abolishes immunoglobulin class switching. Among the other models devoid of parts of the *3′RR* (3), the *c3′RR* model is the only one in which the four core enhancers are present. This model has been instrumental in the conclusion that the core enhancers are the key elements for CSR regulation. It became clear that components of the *3′RR* super-enhancer display specific functions: while CSR is supported by enhancers alone, SHM requires both *3′RR* core enhancers and its palindromic architecture (7,8). Beyond transcriptional control of SHM and CSR, additional levels of regulation occur during late B-cell maturation. Most of Ig gene regulation studies have been so far performed at nucleosomal scale (epigenetic modifications and regulatory transcription of loci and gene segments). The increasing interest for the understanding of gene regulation at the whole nuclei scale raises the necessity to revisit previous models at both supranucleosomal (DNA loops and A/B compartments) and nuclear (chromosome territories and gene positioning) levels (9). A and B subcompartments were revealed by Hi-C data, and in a simplified scheme, gene transcription takes place in euchromatin (A) while heterochromatin (B) prevents it (10). To date, a supranucleosomal level of regulation has been demonstrated in mature and stimulated B cells by describing the dynamics of *IgH* chromatin loops during CSR (11). CSR mechanism involves DNA double-strand breaks (DSBs) at *Sμ* donor and *Sx* acceptor regions (12). Mammalian switch (*S*) regions, located upstream of each constant gene except *Cδ*, consist of 1–10-kb-long highly repetitive G-rich DNA sequences containing clusters of RGYW AID deamination hotspots (13). The processing of AID-induced DSB at *S* donor and acceptor regions is essentially ensured by the non-homologous end joining (NHEJ) pathway (14,15). A typical successful and productive class switch event, also called deletional CSR, deletes the intervening DNA regions located between both DNA DSB and finally replaces the *Cμ* constant gene by one of the six *Cx* constant genes (*IgG3*,*IgG1*,*IgG2a*,*IgG2b*,*IgA*and*IgE*) (16). Mature resting B cells ensure an *IgH* locus configuration ‘prepared for CSR’ by bringing in close contact the *3′RR* and *Eμ*-regions, thereby forming a 50–200 kb chromatin ‘big loop’ that contains all *S* regions. Upon stimulation, this configuration acquires additional contact between the acceptor *S* region involved in CSR and the previously interacting *IgH* enhancers (17–19). In this new layout, *S* donor and *S* acceptor regions are aligned to favor productive deletional CSR. To note, some atypical events can also occur in activated B cells: some of them, named inversional CSR, flip the intervening DNA region located between donor and acceptor S regions. By inverting the *IgH* constant regions located downstream from the *VDJ* exon, these latter events are considered non-productive since they deprive the B cell of its Ig heavy chain (20). The inhibition of DSB response factors impacts the ratio of deletional versus inversional CSR events and promotes long end resection in *S* regions, highlighting the critical role of DNA repair pathway for efficient deletional CSR (20–22). The current model proposes that chromatin loops in Ig gene loci are formed by loop extrusion, a mechanism consisting in loading cohesin complexes at transcribed enhancers or promoters (23–25) followed by chromatin extrusion until cohesin meets convergently oriented CTCF sites (5). In the specific case of *IgH* locus, cohesin is likely to be loaded at enhancers (either *3′RR* or *Eμ* regions) to first initiate the ‘big loop’ between *3′RR* and *Iμ/Sμ* regions at the resting stage. Upon B-cell stimulation, new internal loops are shaped to bring together transcribed *S* regions in order to form the CSR center (CSRC) (18,19), a structure that favors deletional CSR recombination (20).

Recent advances in the understanding of CSR mechanisms at the level of chromatin loops have raised the question of the involvement of *3′RR* components (core enhancers and palindromic structure) in CSR center formation, CSR junction orientation and DNA repair. Here, by taking advantage of two mouse models devoid of either the entire *3′RR* [Δ*3′RR* (6)] or only its palindromic structure [*c3′RR* model (7)], we evaluated by multiple approaches the role of *3′RR* core enhancers in B-cell nuclear organization and *IgH* locus architecture. Interestingly, we showed that, even in the absence of the global *3′RR* palindromic structure, the four core enhancers of this region are necessary and sufficient to pre-organize the *IgH* locus in the nucleus of resting B cell to allow productive successful deletional CSR.

## Materials and methods

### Mice

Eight-to 10-week-old *wt*, Δ*3′RR*, *c3′RR* or Δ*hs5-7* (kindly provided by Barbara Birshtein) mice were bred and maintained in SOPF (Specific and opportunistic pathogen free) conditions at 21–23°C with a 12 h light/dark cycle. The procedures were reviewed and approved by the Ministère de l’Education Nationale de l’Enseignement Supérieur et Recherche, referring to APAFIS#16151-2018071716292105v3.

### Cell culture

Splenocytes were harvested and resting B cells were collected by using the mouse B-cell isolation kit (STEMCELL Technologies). *In vitro* stimulated B cells were collected after 3 or 4 days of culture at 1.10^6^ cells/ml in RPMI 1640 medium supplemented with 10% FCS (Fetal Calf Serum), 2 mM glutamine (Eurobio), 1% Eagle’s non-essential amino acids (Eurobio), 50 U/ml of penicillin–streptomycin (Gibco), 1 mM sodium pyruvate (Eurobio), 129 μM 2-β-mercaptoethanol (Sigma–Aldrich) and 1 μg/ml lipopolysaccharide (LPS EB Ultrapure, Invivogen).

### Immunization

Mice were immunized by intraperitoneal injection of 200 μl of sheep red blood cells (SRBCs), challenged on day 10 and sacrificed on day 18. Splenic B cells were enriched by using CD43-negative selection (Miltenyi).

### Flow cytometry and cell sorting

To sort transitional B cells, B splenocytes were labeled with anti-B220-APC (RA3-6B2 clone, BD 553092) and anti-CD93-BV421 (AA4.1 clone, BD 561990) conjugated antibodies. Transitional (B220^+^/CD93^+^) B cells were sorted on ARIA III apparatus (BD Biosciences). Plasmablasts were labeled with anti-B220-BV421 (RA3-6B2 clone, BD 562922) and anti-CD138-APC (281-2 clone, BD 558626).

### DNA 3D-FISH

DNA 3D fluorescence *in situ*hybridization (FISH) was performed as previously described (26). Briefly, resting and *in vitro* stimulated B cells for 3 days were dropped onto poly-l-lysine-coated slides and fixed with 4% paraformaldehyde for 10 min at room temperature (RT). Fixed cells were washed in PBS (phosphate-buffered saline) baths, permeabilized with pepsin 0.02%/HCl 0.1 M for 10 min at 37°C, washed again with PBS and submitted to post-fixation with 1% paraformaldehyde for 5 min at RT. Genomic DNA and probes were denatured in 70% formamide/2× SSC (saline-sodium citrate solution) at 72 and 95°C for 5 min, respectively, and hybridized overnight at 37°C. DNA probes specific of *IgH* were labeled with dCTP-biotin or dUTP-digoxigenin (Invitrogen); major γ-satellite probe (which highlight heterochromatin) was labeled with dUTP-Alexa Fluor 488 (Invitrogen). Slides were washed in 1× SSC at 72°C for 5 min and then incubated with streptavidin–Alexa Fluor 594 (Molecular Probes) or anti-digoxigenin-rhodamine (Roche) (1/300^e^ in 4× SSC) for 1 h at RT and mounted with Vectashield containing DAPI (Vector Labs). Images were captured with an epifluorescence microscope (NIKON). Optical sections separated by 0.2 μm were captured and stacks were deconvoluted using Huygens software. Analysis of distances (measured from the center of each signal) was performed using Volocity software. Volumetric pixel size was 0.065 μm in *xy* and 0.2 μm in *z* direction.

### ATAC-seq

Ten thousand resting or 3-day *in vitro* stimulated B cells were centrifuged at 500 × *g* at 4°C during 10 min. Cells were incubated in lysis buffer (0.1% Tween 20, 0.1% Nonidet P-40, 0.01% digitonin, 10 mM Tris–HCl (pH 7.4), 10 mM NaCl, 3 mM MgCl_2_) on ice for 3 min and resuspended in 10 μl of transposition mix (1× TD buffer from Illumina, Transposase from Illumina, 0.01% digitonin, 0.1% Tween 20). Tagmentation was performed on a thermomixer at 1000 rpm for 30 min at 37°C. Transposed DNA was cleaned up with minElute Qiagen kit (#28006). Transposed DNA was amplified by PCR using primers Ad1 (5′-AATGATACGGCGACCACCGAGATCTACACTCGTTCGGCAGCGTCAGATGTG-3′) and Ad2.X (5′-CAAGCAGAAGACGGCATACGAGAT**BARECODE**GTCTCGTGGGCTCGGAGATGT-3′). Libraries were sequenced at 2 × 100 bp on NovaSeq 6000.

### ATAC-qPCR

DNA accessibility was quantified in resting B cells by qPCR using the SYBR Hi-ROX kit (Bioline). Accessibility of *Cμ* and *Cγ3* regions was quantified using *Cμ*-Forward (5′-CTTCCCAAATGTCTTCCCCC-3′), Cμ-Reverse (5′-TGCGAGGTGGCTAGGTACTTG-3′), *Cγ3*-Forward (5′-GTTCCCATTAGAAAAATACC-3′) and *Cγ3*-Reverse (5′-CTGCTAGATTCTGCACTGAC-3′). *Cμ* and *Cγ3* regions’ accessibility was normalized to *Slc19a2* gene identified as invariant between our sample, using *Slc19a2*-Forward (5′-TACGGC TTCTTCGCAAACCT-3′) and *Slc19a2*-Reverse (5′-TCGGTCAAGTTCTTGTCGGG-3′), as described in the ‘Analysis of ATAC-seq’ section.

### 3C-HTGTS

3C-HTGTS (high-throughput genome-wide translocation sequencing) was performed as previously described (5). Briefly, 10 million resting or 3-day stimulated B cells were cross-linked with 2% formaldehyde/10% FCS PBS for 10 minutes at RT (Room Temperature) with shaking. Cross-linking was stopped by the addition of glycine at 0.1 M. Cells were then lysed in 50 mM Tris, 150 mM NaCl, 5 mM EDTA and 0.5% Nonide P-40, 1% Triton X-100 supplemented with protease inhibitor (Roche). Nuclei were resuspended in 0.3% sodium dodecyl sulfate for 1 h at 37°C at 900 rpm and then neutralized with Triton X-100 for 1 h. DNA restriction was performed using *CviQ1* restriction enzyme in B buffer (Thermo Fisher) overnight at 37°C, before heat inactivation for 25 min at 65°C. Overnight ligation was performed at 16°C at 300 rpm. Next, DNA was treated by proteinase K and RNase and cleaned by phenol/chloroform. After 3C step, the LAM-HTGTS protocol was performed (27). Briefly, 3C DNA was sonicated using the Bioruptor (Diagenode), by two pulses at low intensity for 20 s; 10 μg of sonicated sample was used for the LAM-HTGTS step. A *3′CBE* (5′biotin-CACTGTCCAGACAGCAAACC-3′), *Iμ/Sμ* (5′biotin-GCAGACCTGGGAATGTATGGT-3′) or *3′RR* (5′biotin-GGACTGCTCTGTGCAACAAC-3′) bait was used for primer extension. These single-stranded DNA fragments were incubated with C1 streptavidin Dynabeads (Invitrogen) overnight at RT and washed with BW buffer (1 M NaCl, 5 mM Tris–HCl, pH 7.4, 0.5 mM EDTA, pH 8.0). A universal I7 adaptor (5′-GCGACTATAGGGCACGCGTGGNNNNNN-3′NH2 and 5′-P CCACGCGTGCCCTATAGTCGC-3′NH2) was ligated prior to the nested PCR performed with *3′CBE*-bait nested primer (5′-ACACTCTTTCCCTACACGACGCTCTTCCGATCT**BARECODE**ACCGGCATGTTCATCAACAC-3′) or *Iμ/Sμ*-bait nested primer (5′-ACACTCTTTCCCTACACGACGCTCTTCCGATCT**BARECODE**ACACAAAGACTCTGGACCTC-3′) or *3′RR*-bait nested primer (5′ACACTCTTTCCCTACACGACGCTCTTCCGATCT**BARECODE**CAAGCTGGGGTCAGAGCATG-3′) and universal I7 reverse (5′-CTCGGCATTCCTGCTGAACCGCTCTTCCGATCTGACTATAGGGCACGCGTGG-3′). After tagged PCR with I7 and I5 Illumina primers, PCR products were cleaned using PCR clean-up kit (Macherey–Nagel) and validated after migration on BioAnalyzer (Agilent). 3C-HTGTS libraries were sequenced at 2 × 300 bp paired-end MiSeq V3 with 20% PhiX. qPCR was performed using *Iμ/Sμ* forward primer in combination with *Iμ/Sμ* reverse primer (5′-CACAAATGTCTCTTTCTCCC-3′) to normalize the data. The specific interaction frequencies were quantified using the *Iμ/Sμ* forward primer in combination with the following reverse primers: *Sγ3* (5′-AGGCCATCACCTTACCTGTG-3′), *Sγ2* (5′-GCGTGAAGAAGACTGCTGCT-3′), *3′RR-hs4* (5′-AATGGGGCTTTCCACGCC-3′) and *3′CBE* (5′-ACCGGCATGTTCATCAACAC-3′).

### LAM-HTGTS

Genomic DNA (gDNA) was extracted from 10 million 4-day stimulated B cells or CD43^−^ B cells sorted from spleen of SRBC immunized mice. LAM-HTGTS was performed as previously described (27) using an *Sμ* bait. Read datasets obtained with MiSeq were analyzed as previously described (28). All sequence alignments were performed against the mouse mm10 genome. Analysis of junction structure was performed using CSReport tool (29).

### Analysis of ATAC-seq

Nextera sequences were trimmed using TrimGalore (30). Only pairs of reads with a minimum length of 55 bp each were reported in the resulting fastqs. The nf-core/atacseq pipeline (28) was used to process the data, with mm10 as the reference genome. Differentially opened chromatin regions from comparisons of different conditions were extracted using DESeq2 (31) from the *DESeqDataSet* previously generated by the pipeline (32).

The normalized counts extracted from the *DESeqDataSet* were used to calculate the coefficient of variation (CV) of each consensus peak. Peaks with coverage greater than or equal to the mean were ranked by increasing CV to find a suitable region for ATAC-qPCR normalization controls among the samples compared. The second consensus peak hitting *Slc19a2* gene from positions 164248151 to 164249868 on chromosome 1 on mm10 genome assembly was selected to design primers for ATAC-qPCR normalization controls. Chromatin A/B compartments were inferred from the consensus peaks of nf-core/atacseq analysis (32) at 4000 kb resolution using an extended version (github trichelab/compartmap repository) of the original R package Compartmap (33).

### Analysis of 3C-HTGTS

Sequencing reads were aligned to the mm10 genome and processed as previously described (27). Each 3C-HTGTS library plotted for comparison was normalized by randomly selecting a number of junctions equal to the total number of junctions present in the smallest library in the comparison set.

### Statistical analysis

Mann–Whitney two-tailed tests were used for statistical analysis using GraphPad Prism software (*P< 0.05, **P< 0.01, ***P< 0.001 and ****P< 0.0001).

## Results

### 3′RR core enhancers maintain *IgH* loci in an active compartment in mature resting B cells

To study the role of *3′RR* core enhancers versus*3′RR* palindromic structure, we compared two mouse models carrying total or partial deletion of the *3′RR* to *wt* mice. We took advantage of the *c3′RR* model (7) carrying only the four core enhancers and the Δ*3′RR* model (6) carrying total deletion of the *3′RR* region (Figure 1A). Such a comparison is strongly relevant to study the role of the palindromic structure in the context of CSR recombination since CSR levels are almost completely restored in the *c3′RR* model compared to the *3′RR* KO mice (7). B-cell subsets examined in these three models were transitional splenic B cells (T) collected by flow cytometry (Supplementary Figure S1A), mature resting B cells (R) and *in vitro*stimulated B cells for 3 days with LPS (S). To avoid any bias related to cell subtypes representation in bulk culture at day 3, we verified the percentage of CD138-positive cells after LPS stimulation in the three models. As expected, the percentage of newly *in vitro* generated plasmablasts in the presence of LPS was comparable between models (Supplementary Figure S1B).

By using ATAC-seq, we determined, with a high-throughput approach, chromatin accessibility across the genome in resting and *in vitro*stimulated B cells from *wt*, *c3′RR* and Δ*3′RR* mice. Reproducibility between samples was verified by a principal component analysis (Supplementary Figure S2A). First, we confronted the percentage of A (euchromatin) and B (heterochromatin) compartments between mutants and we did not observe any significant change either in resting B cells or in *in vitro* stimulated B cells (Figure 1B). These data show that deletion of the *3′RR* (Δ*3′RR* model) or the palindromic structure (*c3′RR* model) does not significantly impair global A/B compartmentalization in B cells. Second, we compared differentially opened (DO) regions in resting and stimulated B cells of all models and identified many regions (Supplementary Table S1A) that we suspected to be DO in response to the B-cell activation program by LPS. To verify this point, we performed an enriched pathway analysis and found in each genotype the same top 12 enriched pathways consistent with lymphocyte/B-cell activation (Supplementary Figure S2B). Third, we also compared either the resting or the activated stage of each genotype: in this case only a few DO regions (<330) were identified (Supplementary Table S1B), suggesting that genome-wide accessibility is not affected by partial or total deletion of the *3′RR*. Afterward, we focused exclusively on the *IgH* locus; we observed a decrease of its accessibility in resting B cells from Δ*3′RR* mutants in comparison to *wt* (Figure 1C). To confirm this trend, we quantified ATAC-seq normalized read count in three distinct regions across the *IgH* locus (R1: around *Eμ/Sμ*; R2: constant regions; and R3: *3′CBE*; whose coordinates are available in Supplementary Table S2). Chromatin accessibility was significantly decreased in constant regions and a similar downward trend was observed in *Sμ* and *3′CBE* regions in the Δ*3′RR* mutant. In contrast, chromatin accessibility in all *IgH* tested regions was recovered upon reintroduction of the core enhancers (Figure 1C). We confirmed ATAC-seq results by performing qPCR in *Cμ* and *Cγ3* regions. To normalize qPCR, we identified the Scl19a2 gene on chromosome 1 as an open region with low variability between samples. We noticed, by qPCR, a significant decrease in *IgH* accessibility for the *Cγ3* region in *Δ3′RR* resting B cells (Supplementary Figure S2C). As expected, we found that *c3′RR* resting B cells harbor an accessibility profile similar to the *wt*, demonstrating that core enhancers are sufficient to maintain *IgH* loci in an open chromatin conformation (Figure 1C and Supplementary Figure S2C). The same trend of changes in *IgH* accessibility was observed in LPS-stimulated cells by ATAC-seq and qPCR: in the Δ*3′RR* model the *IgH* locus is less accessible whereas the reintroduction of the *3′RR* core enhancers tends to restore the accessibility of the locus (Figure 1D and Supplementary Figure S2C).

**Figure 2.**
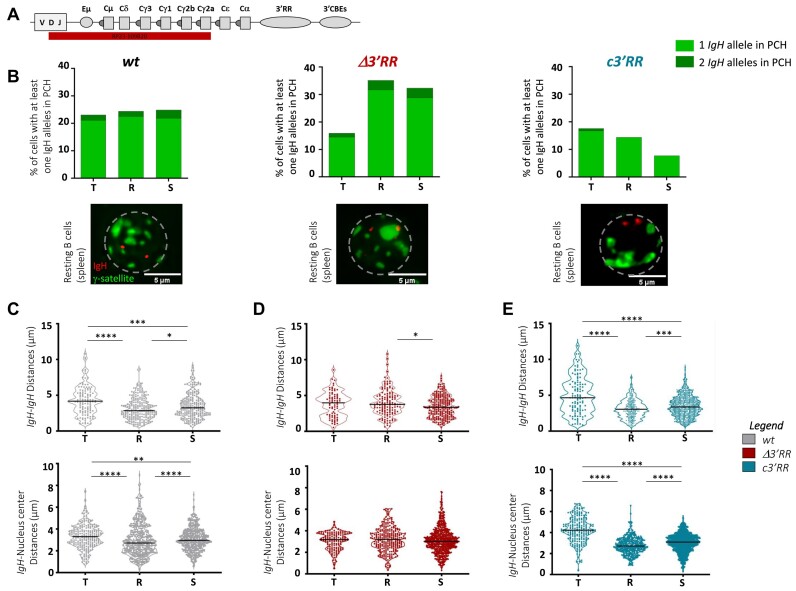
*3* *′* *RR* core enhancers are sufficient to maintain *IgH* loci close together and close to the center in mature resting B cells. (**A**) Localization of the RP23-109B20 FISH probe on the murine *IgH* locus is shown in red. (**B**) Bargraphs represent the percentage of *IgH* loci located into pericentromeric heterochromatin (PCH, stained with γ-satellite major probe) evaluated in B-cell subsets of *wt* and mutants mice by DNA 3D-FISH experiments: in transitional (T) (*n* = 3 *wt* mice/116 nuclei), (*n* = 3 Δ*3′RR* mice/75 nuclei) and (*n* = 2 *c3′RR* mice/108 nuclei), in resting (R) (*n* = 3 *wt* mice/232 nuclei), (*n* = 3 Δ*3′RR* mice/136 nuclei and *n* = 3 *c3′RR* mice/199 nuclei) and in *in vitro* stimulated (S) (*n* = 4 *wt* mice/239 nuclei, *n* = 4 Δ*3′RR* mice/249 nuclei and *n* = 3 *c3′RR* mice/740 nuclei). Light and dark green boxes correspond to the percentage of cells harboring one or two *IgH* allele in PCH, respectively. A representative nucleus at the resting stage is shown for each mouse model (*IgH* loci in red/PCH in green, scale bars: 5 μm). (**C**) Violin plots represent raw *IgH*to*IgH* interallelic distances (top) and *IgH to* nucleus center distances (bottom) in μm from transitional to stimulated B-cell stages in *wt*. (**D**) Same as in panel (**C**) for Δ*3′RR* mice. (**E**) Same as in panel (**C**) for *c3′RR* mice Top: each point on the violin plot represents a single measurement between the two *IgH* alleles in a single B-cell nucleus. Bottom: each point on the violin plot represents a distance measured between one *IgH* allele and the nuclear center. *P*-value was determined using a two-tailed Mann–Whitney test; only significant differences are indicated (*P< 0,05; **P< 0,01; ***P< 0001; ****P< 0,0001).

Subsequently, we assessed addressing of *IgH* loci to pericentromeric heterochromatin (PCH) within B cells nuclei by the DNA 3D-FISH approach using an *IgH* fluorescent probe (RP23-109B20) encompassing a region lying from *D_H_* cluster to *Cγ2a* segment (Figure 2A) and a probe (major γ-satellite) specific to PCH as the ‘inactive zone’. For the need of the study, we made the choice to include transitional B cells in order to analyze the kinetics of *IgH* allele nuclear localization occurring in developing mature B cells. In a *wt* context, in all three cell types tested, ∼20% of cells have at least one *IgH* allele in the PCH, with a minor proportion of cells having both *IgH* alleles in PCH (Figure 2B, left). In Δ*3′RR* mice, around 15% of *IgH* alleles are localized in PCH at transitional stage, a situation closed to that observed in *wt* mice. In contrast, in resting B cells devoid of *3′RR*, the proportion of B cells harboring at least one *IgH* allele colocalized to PCH reaches 35%; moreover, this population includes a higher proportion of cells for which both alleles localized to PCH (Figure 2B, middle). This configuration is maintained in *in vitro* stimulated B cells. Surprisingly, when only *3′RR* core enhancers are reintroduced in B-lineage cells (*c3′RR* model), PCH localization of *IgH* alleles returns to virtually the same profile as observed in *wt* (Figure 2B, right). Overall, DNA 3D-FISH and ATAC-seq experiments showed a global decrease of *IgH* loci accessibility in resting B cells and in *in vitro* stimulated B cells from Δ*3′RR*. In contrast, *c3′RR* B cells are in an open chromatin configuration nearly similar to the *wt* context. Taken together, these results show that in *wt* resting B cells, *IgH* loci were widely in an open chromatin compartment and that PCH addressing is governed solely by *3′RR* core enhancers, and not by palindromic structure. These findings point out a crucial role of the *3′RR* core enhancers in regulating the nuclear organization of mature resting B cells.

### 3′RR core enhancers are sufficient to regulate *IgH* loci position dynamics in nuclei of mature B cells

Using DNA 3D-FISH, we also investigated the position of *IgH* loci within nuclei of transitional, mature resting and *in vitro* stimulated B cells from the three mouse models. We measured the *IgH* interallelic distance as well as the distance between *IgH* loci and nuclear center at each stage of differentiation cited above. In the *wt* context, we observed dynamic changes in the nuclear position of *IgH* alleles when B cells progress through maturation and *in vitro* stimulation: first, *IgH* loci move closer to each other (*P*< 0.0001) during the transitional to mature resting stage switch, and then move away during *in vitro* stimulation (*P*= 0.0122) (Figure 2C, top). In agreement with interallelic data, the same dynamic is observed when we measured the distances between *IgH* loci and the nuclear center in *wt* mice: during the shift from transitional to resting stage, *IgH* loci move closer to the center of nucleus (*P*< 0.0001), while they move away from the center upon *in vitro* stimulation (*P*< 0.0001) (Figure 2C, bottom). In contrast, *IgH* loci lost their dynamic positioning observed in the *wt* context in B cells deficient for the entire *3′RR* (Δ*3′RR* model): both *IgH* interallelic distance (Figure 2D, top) and relative position to the center of the nucleus remain constant at transitional, resting and stimulated stages (Figure 2D, bottom). Interestingly, the reintroduction of *3′RR* core enhancers (*c3′RR* model) alone rescued normal dynamics: *IgH* loci move toward each other during transitional to resting stage evolution (*P*< 0.0001) and then move away upon *in vitro* stimulation (*P*= 0.0004) (Figure 2E, top). Comparably, in nuclei from *c3′RR* resting B cells, *IgH* loci are closer to the center than in nuclei from transitional B cells (*P*< 0.0001); *IgH* loci also move away from the center during stimulation (*P*< 0.0001) (Figure 2E, bottom). These results pinpoint a relocalization of *IgH* loci during late B-cell maturation and highlight the crucial role carried by the *3′RR* in these dynamic nuclear events. More precisely, our study shows that, within the large *3′RR*, the core enhancers ensure by themselves the dynamic moves of *IgH* loci in B-cell nuclei. The investigation of *IgH* nuclear localization across transitional, resting and stimulated B-cell subsets indicates that major differences are evident at resting B-cell stage. This suggests that the nuclear pre-organizing function of the *3′RR* core enhancers is a feature of this specific stage.

### 3′RR core enhancers enable *IgH* loop conformation in mature resting and stimulated B cells

**Figure 3.**
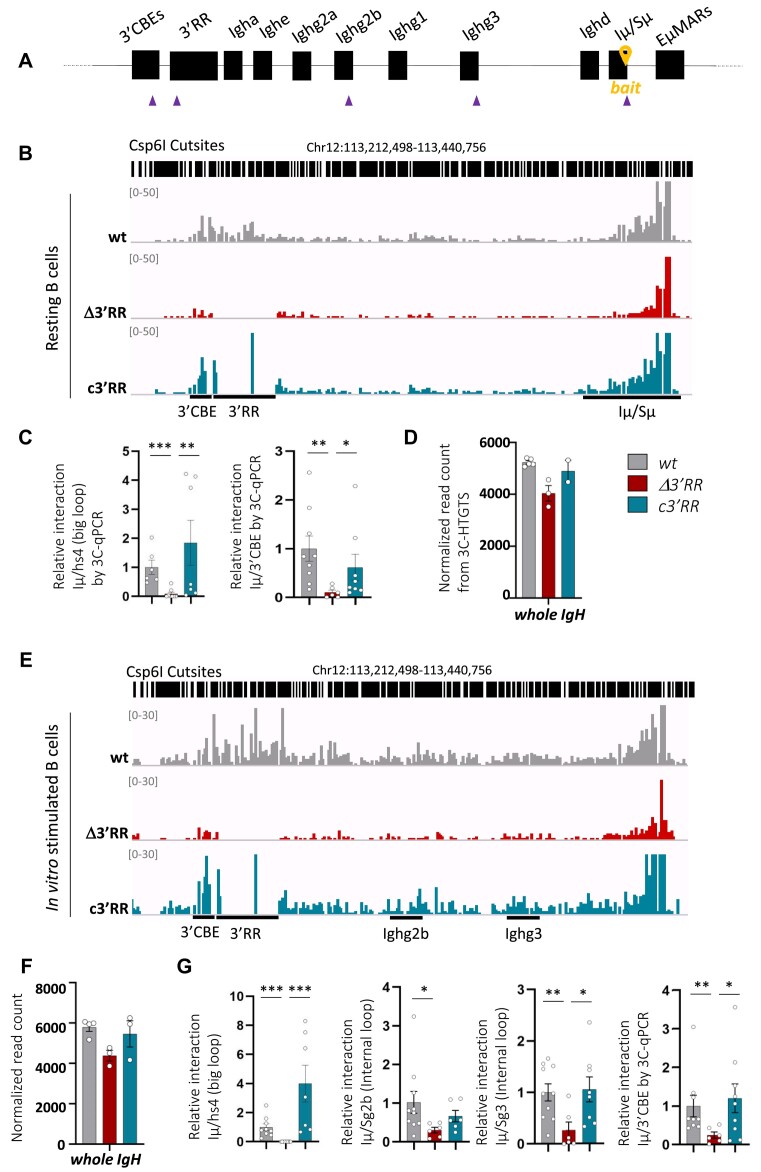
*3′RR* core enhancers are mandatory for *IgH* big loop and internal loop formation at resting and stimulated B-cell stages. (**A**) Representation of the murine *IgH* locus, the 3C-HTGTS bait and the 3C-qPCR primers are indicated by a yellow location pin and purple triangles, respectively. (**B**) Representative bedgraphs, visualized by IGV tool, showing chromatin interactions in splenic resting B cells from independent *wt* (*n* = 5), Δ*3′RR* (*n* = 3) and *c3′RR* (*n* = 2) mice. Bedgraphs were normalized to the smallest sample. (**C**) The *Iμ-Sμ/3′RR-hs4* and *Iμ-Sμ/3′CBE* loop interactions were precisely quantified by 3C-qPCR from independent *wt* (*n* = 6–9), Δ*3′RR* (*n* = 6–8) and *c3′RR* (*n* = 7–8) mice. (**D**) Quantification of normalized read count within the entire *IgH* locus in splenic resting B cells from *wt* and mutant mice. (**E**) Same representation as in panel (**B**) in *in vitro* LPS-stimulated B cells from independent mice of *wt* (*n* = 4), Δ*3′RR* (*n* = 3) and *c3′RR* (*n* = 3) models. (**F**) The *Iμ/3′RR-hs4*, *Iμ/Sγ3, Iμ/Sγ2b* and *Iμ/3′CBE* interactions were precisely quantified by 3C-qPCR from independent mice of *wt* (*n* = 9–10), Δ*3′RR* (*n* = 6–9) and *c3′RR* (*n* = 6–8) models. (**G**) Same representation as displayed in panel (**D**). Bedgraph scale was adjusted to show interactions of interest. Error bars represent SEM. *P*-value was determined by a two-tailed Mann–Whitney test; only significant differences are indicated (*P< 0,05; **P< 0,01; ***P< 0001).

As a supplemental level of regulation, chromatin loop formation appears as an essential mechanism to orchestrate gene expression in a defined TAD. Within the TAD encompassing the *IgH* locus, loop extrusion has been recently proposed as a critical mechanism enabling specific chromatin loops that facilitate recombination events taking place in bone marrow and periphery (18,34). However, to date, the regulation of loop formation in the context of CSR is not fully described. Our mouse models allowed us to assess the respective involvement of components of the *3′RR* for *IgH* locus looping in B cells undergoing CSR. To achieve this point, we quantified chromatin loops from bait to prey (Supplementary Table S3A for prey coordinates) by high-resolution 3C-HTGTS (18) in resting and LPS-stimulated B cells from *wt* and mutant mice. Two complementary viewpoints (or baits) were used to assess loop formation: one is located within the *Iμ/Sμ* region (named *Iμ/Sμ* bait) (Figure 3A and Supplementary Figure S4A) (20) and the other one, called *3′CBE* bait, conserved among the three models, is located between *hs5* and *hs6* insulators within the 3′ *IgH* TAD border (Supplementary Figure S4A).

When performed in *wt* resting B cells, 3C-HTGTS conducted with *Iμ/Sμ* bait showed long-range contacts between *Iμ/Sμ* and the *3′RR* region (Figure 3B, bedgraphs, Supplementary Figure S3B for individual mice and Supplementary Table S3B), this confirmed that the *3′RR* super-enhancer is an essential distant partner of the *Iμ/Sμ* region. Consistent with both pioneer studies based on chromosome conformation capture (17) and the more recent studies built on high-throughput techniques (18), these data reflect the first level of *IgH* locus folding (also called ‘big loop’). As a logical consequence of the absence of the prey, Δ*3′RR* resting B cells were unable to perform the big loop (Figure 3B, bedgraphs, Supplementary Figure S3B for individual mice and Supplementary Table S3B). Strikingly, in *c3′RR* resting B cells, chromatin *IgH* big loops were restored and mainly involved *hs1.2* and *hs4* elements in the absence of the palindromic structure (Figure 3B, bedgraphs, and Supplementary Figure S3B for individual mice and Supplementary Table S3B). Accurate quantification by 3C-qPCR shows that loop interactions between *Iμ/Sμ* and *3′RR* regions were fully restored by reintroduction of 3′RR core elements. (Figure 3C, left).

In contrast with long-range interactions, local interactions between the *Iμ/Sμ* bait and its downstream region were maintained in the absence of *3′RR*, showing that this region is not involved in local loop formation (Figure 3B, bedgraphs, Supplementary Figure S3B for individual mice and Supplementary Table S3B). Notably, no other distant interaction taking place within the *IgH* locus was detected (Figure 3D); this clearly indicate that the decrease of long-range (with the *3′RR*) interactions from *Iμ* was not compensated by any new interaction inside the locus. Moreover, the absence of the *3′RR* did not favor interaction outside the TAD encompassing the *IgH* locus (*eS* region), a phenomenon previously described in the mouse model devoid of the *3′CBE* region in which the *IgH* TAD boundary is disrupted (5) (Supplementary Figure S3B and Supplementary Table S3B). Interestingly, absence of the *3′RR* reduced the interaction between *Iμ/Sμ* and *3′CBE* regions, while reintroduction of the *3′RR* core enhancers fully restored such interaction (Figure 3B and C, right, Supplementary Figure S3B for individual mice and Supplementary Table S3B). Overall, the core enhancers of the *3′RR* are sufficient to establish loop interaction between *3′CBE* and *Iμ/Sμ* regions. Main *IgH* loop interactions described above were validated by using the *3′CBE* bait located downstream from 3′ super-enhancer (Supplementary Figure S4A). This reverse viewpoint also showed similar differences in chromatin loop patterns in *wt*,Δ*3′RR* and *c3′RR* models, confirming data obtained with *Iμ/Sμ* viewpoint (Supplementary Figure S4B).

For the first time, these results demonstrate that (i) the *3′RR* core enhancers are necessary and sufficient to provide the first level of folding, the so-called *IgH* ‘big loop’, in resting B cells and (ii) the ‘inverted repeated regions’ maintaining the quasi-palindromic organization of the *3′RR* are not essential for such loop.

The occurrence of chromatin loops was further investigated in *in vitro* stimulated B cells with LPS for 3 days. In the *wt* context, DNA loops detected with *Iμ/Sμ* viewpoint revealed, in addition to the existing ‘big loop’ structure, the occurrence of new interactions with internal *Sγ3* and *Sγ2b* regions that normally undergoes germline transcription upon LPS stimulation (Figure 3E, Supplementary Figure S3C for individual mice and Supplementary Table S3C). These data reflects the physiological *IgH* ‘internal loops’ formed with targeted switch regions in the CSRC (18). Impact of the entire *3′RR* deletion (Δ*3′RR* model) is even greater at this stage than in resting B cells, the *IgH* big loop is still not detectable and a drastic decrease in contact frequencies between *Iμ/Sμ* and *Sγ3* or *Sγ2b* regions is observed (Figure 3E, Supplementary Figure S3C for individual mice and Supplementary Table S3C). As in resting B cells, the abrogation of long-range interactions upon *3′RR* deletion was not compensated by any random interactions within the *IgH* locus (Figures 3E and F), and the local interactions within *Sμ* are maintained (Figure 3E, Supplementary Figure S3C for individual mice and Supplementary Table S3C). In contrast, by using *Iμ/Sμ* bait in LPS-stimulated B cells from *c3′RR* model, the *IgH* big loop was still detected, mainly through the *hs1.2* and *hs4* enhancers (Figure 3E, Supplementary Figure S3C for individual mice and Supplementary Table S3C). An accurate quantification by 3C-qPCR proves that loop interactions between *Iμ/Sμ* and the *3′RR* were abrogated upon complete deletion and fully recovered by the reintroduction of core elements (Figure 3G, left). On the other hand, the frequency of contact between *Iμ/Sμ* and *Sγ3* or *Sγ2b* regions occurring in the *c3′RR* model was almost at the same level as in *wt* mice (Figure 3E and G, middle, Supplementary Figure S3C for individual mice and Supplementary Table S3C). As in resting B cells, loops involving the *Iμ/Sμ* and *3′CBE* regions taking place in activated cells were decreased in the absence of the *3′RR* and recovered by addition of the four enhancers (Figure 3E and G, right, Supplementary Figure S3C for individual mice and Supplementary Table S3C). 3C-HTGTS performed with the second viewpoint (*3′CBE* bait) confirmed all these findings. Indeed, high levels of interaction between the bait and the *Iμ/Sμ* region were detected in *wt* and *c3′RR* mice as well as internal loops involving the *Sγ3* or *Sγ2b* regions (Supplementary Figure S4C). As expected, there was still a defect of interactions between *Iμ/Sμ* and target switch regions in stimulated Δ*3′RR* B cells (Supplementary Figure S4C).

These data support several hypotheses regarding the establishment of *IgH* loops during B-cell activation. First, it seems quite clear that the *3′RR* core enhancers alone (without their flanking inverted repeated DNA regions) are sufficient in both resting and stimulated B cells for the formation of multiple *IgH* chromatin loops: the ‘big loop’ (*Iμ/3′RR*), the internal CSR loops (*Iμ/S* acceptor) and the *Iμ/3′CBE* loop, in both resting and stimulated B cells. Second, formation of the big loop at resting stage is a prerequisite for the formation of internal loops and consequently CSR in activated B cells. The internal loop defect identified in the Δ*3′RR* model by 3C-HTGTS reflects the failure to form the CSRC; this is consistent with the strong CSR defect associated with this deletion (35).

To confirm the role of the *3′RR* core enhancers as organizers of *IgH* chromatin loops, we decided to interrogate involvement of the *IgH 3′CBE*, a major part of the TAD boundary, in loop formation. To this end, we performed 3C-HTGTS on a mouse model carrying a deletion of most of the *3′CBE* region (deletion from *hs5* to *hs7*) (36) with a bait located immediately downstream from the *hs4* distal enhancer (bait *3′RR*) but conserved upon deletion of the entire *3′RR* (Supplementary Figures S5A and B). In this model named Δ*hs567*, we still observed the formation of the *IgH* big loop in resting B cells as well as internal CSR loops in stimulated B cells. Taken together, these results indicate that the *3′CBE* region is not involved in the formation of *IgH* chromatin loops (Supplementary Figures S5C and D).

### 3′RR core enhancers are required for productive successful deletional CSR mechanism

**Figure 4.**
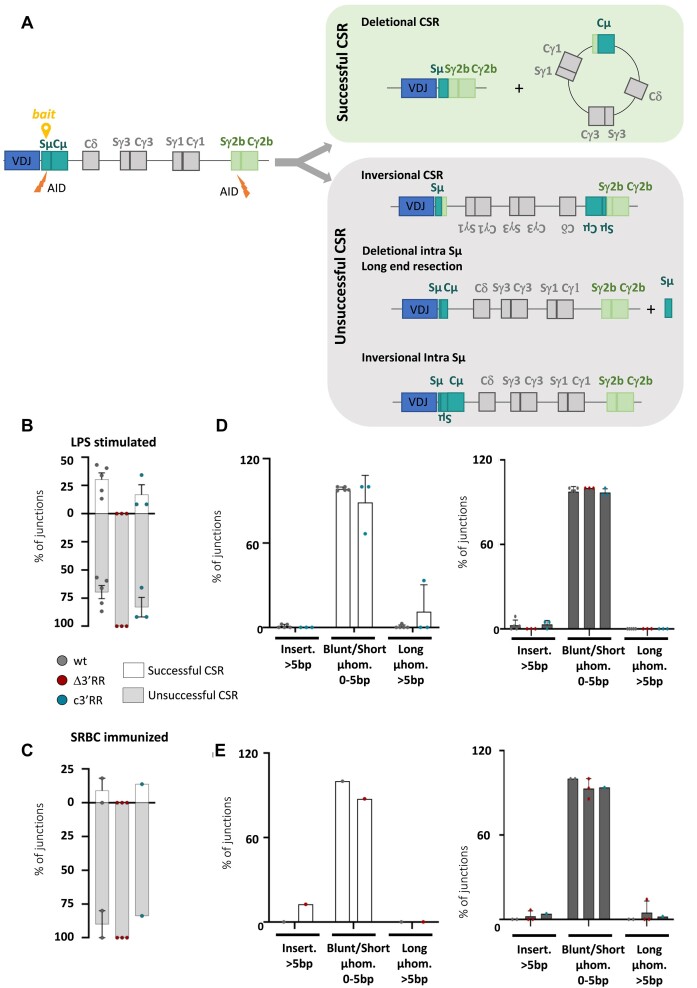
Deletional CSR in stimulated B cells requires *3′RR* core enhancers. (**A**) Scheme representation of recombination events leading to successful and unsuccessful CSR. Successful CSR corresponds to a classical deletional event involving *Sμ* and target switch regions (*Sγ3* and *Sγ2b* in LPS stimulation condition and *Sγ1* in SRBC immunized mice), leading to the excision of the intermediate DNA segment. Unsuccessful CSR events include inversional CSR, deletional intra-*Sμ* and inversional intra-*Sμ* recombination events. The location of the *Sμ* bait for LAM-HTGTS is indicated by a yellow location pin. (**B**) Bargraphs represent the proportion of successful CSR (white bars) and unsuccessful CSR (gray bars) determined by LAM-HTGTS in LPS-stimulated B cells from independent *wt* (*n* = 5), Δ*3′RR* (*n* = 3) and *c3′RR* (*n* = 3) mice. (**C**) Same as in panel (**B**) for splenic B cells sorted from SRBC immunized mice of *wt* (*n* = 2), Δ*3′RR* (*n* = 3) and *c3′RR* (*n* = 1) models. (**D**) Features of CSR junctions, analyzed with CSReport (29), in successful (right, white bars) and unsuccessful (left, dark gray bar) events obtained in LPS-stimulated splenic B cells. (**E**) Same as in panel (**D**) for splenic B cells sorted from SRBC immunized mice. Error bars represent SEM.

Productive successful CSR events are the result of deletional recombination events that occur by joining the correctly juxtaposed *Sμ* donor and *Sx* acceptor regions in such a way as to achieve deletion of intermediate DNA sequences (Figure 4A, green box). In some cases, the recombination event breaks and ligates the large intermediate DNA segment between the two *S* regions in an inverted manner (20). This process, therefore called inversional CSR, leads to an inversion of the *IgH C* gene and prevents expression of a functional *Ig* heavy chain (Figure 4A, gray box, top). Shorter deletional or inversional recombination events also take place within the *Sμ* region, reflecting the strong targeting of the donor *S* region by AID (Figure 4A, gray box, middle and bottom). Inversional CSR and intra *Sμ* deletion both lead to unsuccessful CSR. In our mutants, we questioned the diversity of recombination events in the *IgH* locus by LAM-HTGTS using a bait localized within *Sμ* (Figure 4A) as previously described (19,23). This high-throughput method, combined to a dedicated analysis pipeline, provides precise recombination junctions; the coordinates used to determine the prey are indicated in Supplementary Table S4. After *in vitro* stimulation with LPS for 4 days, we compared the occurrence of CSR junctions from *Sμ* to *Sγ3* and *Sγ2b* and intra-*Sμ* recombination (Supplementary Figure S6A). In line with previously published data (8,37), LAM-HTGTS experiments showed that CSR and intra-*Sμ* recombination are quite similar in *wt* and *c3'RR* models, whereas CSR is drastically decreased in favor of intra-*Sμ* recombination in the absence of the entire *3′RR* (Supplementary Figure S6A). In addition, deletional CSR predominated over inversional CSR in *wt* as well as in the *c3′RR* model. In contrast, residual CSR events were made by inversion in the *3′RR-*deficient model (Supplementary Figure S6B). In this same model, in which intra-*Sμ* recombination prevails, an increase in inversion events was also observed within the *Sμ* region (Supplementary Figure S6C). These results suggest that the *3′RR* core enhancers are sufficient for proper orientation of recombination events. To objectively account for all types of recombination events occurring at *IgH* locus, we distinguished two groups: successful CSR junctions, as a result of productive deletional CSR events (Figure 4A, green box), and unsuccessful CSR junctions, including any recombination that occurred at the locus without producing productive CSR (i.e. inversional CSR, inversional and deletional intra-*Sμ* recombination) (Figure 4A, gray box). In the *wt* context, ∼30% of the junctions were typical to successful CSR deletional mechanism (Figure 4B). The same trend was observed in the *c3′RR* model (Figure 4B). To verify that these observations were not restricted to *in vitro* conditions under LPS stimulation, we sought to evaluate CSR-related recombination events *in vivo* under antigen challenge. We thus performed LAM-HTGTS in splenic B cells sorted from SRBC immunized mice and analyzed the resulting junctions. Again, confirming and strengthening the relevance of our previous data, we found the same bias between successful and unsuccessful CSR events in the Δ*3′RR* mutant, while reintroduction of core enhancers restores a normal ratio (Figure 4C and Supplementary Figure S6D–F). It is widely accepted that detailed analysis of junctions provides reliable information about the repair pathway recruited for CSR event (21,29). Therefore, we analyzed the structural features of both successful and unsuccessful CSR junctions, in *in vitro* LPS-stimulated B cells and in splenic B cells sorted from SRBC immunized mice. In both groups of junctions from all mouse models, we found that the vast majority of junctions have direct joints (blunt) or contain short deletions or microhomologies (Figure 4D and E). As all junctions show features of the NHEJ pathway, the typical pathway involved in normal CSR, our data clearly indicate that the joining process is not affected by partial or total deletion of the *3′RR*. Our data suggest that the 3′RR, through its core enhancer elements, provides an optimal locus conformation to drive the class-switching process toward successful deletional recombination; furthermore, this 3′RR-mediated conformation acts independently on DNA repair factors.

## Discussion

Our study highlights an additional function of the *IgH 3′RR* in the high-order organization of *IgH* locus chromatin in B cells. Through a detailed analysis at different levels of nuclear organization, we showed that the *3′RR* core enhancers are essential and sufficient for pre-organizing *IgH* allele settings in the nuclei of resting B cells to efficiently prepare for major recombination events such as CSR. First, we show that the *3′RR* core enhancers are critical for sustaining *IgH* loci within a transcriptionally active compartment. Second, we show that these regulatory elements display a topological regulatory function in the nucleus of resting B cells by maintaining the *IgH* loci close to each other and to the nuclear center in an active compartment. Third, we show that core enhancers of the *3′RR* are essential for forming the *IgH* big loop in resting B cells as well as the CSR-related internal loops in activated cells. Our current study suggests a mechanistic model in which the *3′RR* core enhancers themselves provide, in resting B cell nucleus, the necessary 3D conformation of *IgH* as a prerequisite for a productive successful deletional CSR process in activated cells (refer to the graphical abstract). Thus, this study also clarifies that the singular palindromic structure of the *3′RR* region does not contribute to any nuclear organization function identified for the *3′RR* core enhancers.

Regarding the colocalization of *IgH* loci with heterochromatin in murine *wt* B cells, it has been acknowledged that only a small proportion of cells contain one *IgH* allele in the PCH, although its location in *in vitro* stimulated B cells is still debated in the light of discordant studies published by the Skok and De Laat groups (38,39). Skok’s group suggests that the unproductive *IgH* allele colocalizes with PCH and replicates later (38), thereby implying that allele exclusion is determined by its nuclear location. In contrast, the De Latt group proposes that biallelic *IgH* transcription is the norm in mature B cells (39). Our study supports this claim by showing that, in almost all *in vitro* activated *wt* B cells, both *IgH* loci remain in euchromatin. These findings are supported by additional studies arguing that CSR events target the two *IgH* alleles (14). Our data also indicate that *IgH* nuclear positioning in an active chromatin compartment is already established in transitional B cells and is maintained both in resting and *in vitro*activated cells. So far, the biological relevance of *IgH* nuclear positioning in mature B cells and beyond (resting, activated and plasma cells) is poorly explored; one study in plasma cells described a pronounced cohabitation between *IgH*, *Igk* and *IgJ* at the nuclear periphery, suggesting a function in nuclear export of Ig transcripts (40). While a conclusive link between *IgH* locus positioning and CSR events cannot be made in our study, our models demonstrated that B cell activation induces dynamic movements of *IgH* loci driven by *3′RR* core enhancers.

Comparison of splenic B-cell subsets in our mutant models revealed that the *3′RR* starts acting at the resting stage to prevent the positioning of *IgH* alleles into the repressive nuclear compartment, and our study further specified that the core enhancers of this large region are the main intervening elements in this process. Consistent with a heterochromatin exclusion function, ATAC-seq data in our models also support the respective role of the *3′RR* and its core enhancers in maintaining global accessibility of the *IgH* locus. Indeed, the recovery of consistent *IgH* accessibility provided by the reintroduction of core enhancers is in agreement with the restoration of germline transcription previously described in this context (7), whereas disruption of the entire *3′RR* reduced both accessibility (this study) and germline transcripts (6). One hypothesis to explain the reduced *IgH* accessibility upon *3′RR* deletion could be the local recruitment or direct binding of chromatin regulatory factors mediated through the *3′RR*. In fact, the *3′RR* enhancer has been shown to recruit BRG1 (also known as SMARCA4), a member of the SWI/SNF chromatin remodeling complex, which is known to open chromatin and activate transcription when bound to enhancer elements. Initially described to bind the *3′RR* in immature B cells (41,42), BRG1 has more recently been found to bind this same region in mature B cells, especially during germinal center formation (43). Another candidate is the ‘chromatin reader’ ZMYND8, which is also able to bind the *3′RR* and whose deletion in B cells leads to the same CSR defect as that observed in the absence of the *3′RR* (44). The potency of BRG1 and ZMYND8 to regulate the *IgH* locus at the supranucleosomal scale is a remaining question.

Considering the multiple and successive loops taking place in the *IgH* locus in mature B cells, our study specified that, within the *3′RR*, core enhancers are the critical elements required for the establishment of such loops in both resting and *in vitro* stimulated cells. Indeed, our study validates the importance for resting B cell to pre-establish the *Sμ/3′RR* big loop, as suggested by pioneer studies driven by the Kenter group (17). We interpret that such a specific conformation could serve several interrelated functions (i) to optimize enhancer/promoter interactions, (ii) to preconfigure the *IgH* locus for CSR and (iii) to maintain CSR targeted regions in a conformation enabling deletional recombination.

First, considering enhancer/promoter interactions, it could be proposed that the *Sμ/3′RR* big loop may initially be mandatory to provide and maintain a consistent level of transcription of the heavy chain in resting B cells, given its proximity to the promoter of the rearranged *V_H_* segment. Indeed, several studies have highlighted the importance of the distal *3′RR hs4* element in promoting the transcription of the rearranged heavy chain in resting B cells and consequently modulating BCR expression at this stage (35,45–47). While these studies proposed that this transcriptional effect was the result of direct enhancer/promoter contact, our present data support such a hypothesis by showing disruption of the *IgH* ‘big loop’ in the absence of the *3′RR* and restoration of the structure by adduction of enhancer elements. The supramolecular Mediator complex could also be involved in loop regulation, especially through Med1 and Med12 (48,49). The Med1 subunit has been shown to activate transcription of the *IgH* locus, particularly at switch acceptor regions and to bind to the *hs1.2* and *hs4* enhancers. The absence of Med1 induced a 2-fold decrease in CSR, an effect potentially linked to the reduction of internal loop formation between *Eμ* and *Sγ2b* upon LPS stimulation (48). Conversely, Med12 knockdown (KD) led to a reduction of AID-induced breaks but also reduced *Eμ/3′RR* big loop and *Sμ/Sα* internal loop formation. These effects observed in the absence of Med12 may reduce the *3′RR* activity, as evidenced by the decrease of the active histone mark H3K27Ac in *3′RR* super-enhancers and *3′RR* eRNAs transcription (49). Finally, the RNA polymerase II pausing and elongation factor Spt5 could also be involved by regulating the *IgH* big loop and internal loops and consequently the CSR recombination through *3′RR* activation; in this case transcription of the *3′RR* itself could be considered as a regulatory loop mechanism since Spt5 deletion impaired this process (50). Exploring roles of the multiple *3′RR* binding elements, especially at core enhancer level, strengthens our findings that *3′RR* core enhancers are essential for the *IgH* locus to display the prepared conformation to undergo CSR. Moreover, our results exemplify the pivotal role of *3′RR* as a platform of factors recruitment, at several levels: *IgH* locus conformation, accessibility and transcription. Since GLT of switch regions is a prerequisite for AID targeting (51,52), it is somehow questionable whether *S* regions transcription and AID recruitment could be involved in loop conformation in B cells during CSR. However, the fact that AID is normally induced in our mouse models (Δ*3′RR* included) suggests that AID expression *per se* is not sufficient to drive *IgH* long range interactions. Conversely, important studies using either 3C-HTGTS (18) and Hi-C (53), describe that *IgH* big and internal loops normally occur in murine primary B cells or CH12 cells in the absence of AID suggesting that the deaminase is not required for *IgH* loops. Regarding the function of GLT in *IgH* loops, the Vian study proposes that the energetics of *IgH* cohesin loops do not need transcription to progress and also showed that once loops are established, their stability does not require transcription anymore (54).

In addition to this first function, our study emphasizes recent studies from the Alt group, which proposed that the *Sμ/3′RR* big loop acts as a prerequiste for the occurrence of *IgH* internal loops that favor CSR in activated B cells (18). Indeed, our study showed that the absence of the *3′RR* can no longer support the appearance of any significant *IgH* loop in both resting and *in vitro*-stimulated B cells. The restoration of both ‘big’ and ‘internal’ loops upon reintroduction of the core *3′RR* enhancers supports the idea that such regulatory elements have a key function in preparing the locus for CSR. Finally, our study strongly suggests that the formation of internal loops in the *IgH* locus depends on the *3′IgH* enhancers and is essential for the productive CSR events in which deletion of intervening DNA regions occurs. Indeed, an increase in atypical CSR events has previously been reported in the case of inhibition/deletion of specific factors involved in the DNA damage response or NHEJ (20–22); however, our data suggest that the normal NHEJ pathway could also support unsuccessful CSR in the absence of components of the *3′RR*. It could then be proposed that the unsuccessful CSR events identified in our Δ*3′RR* mutants are related to the impairment of chromatin looping rather than the recruitment of DNA repair factors.

Our data contradict, at least in part, the recent statements of the Pavri group, performed in AID-deficient B cells, who hypothesized that both the *IgH* big loop and internal loops are mainly mediated by the *3′CBE* region and not by the *3′RR* region (53). Our study, deciphering steady-state loops in AID-competent models, provides evidence that such loops cannot take place in a locus containing the *3′CBE* but lacking *3′RR* elements. A difference between the two studies is the technical approaches: while our 3C-HTGTS detects robust interaction between two partners, the Tri-C method reports multiway interactions. Pavri’s study identified a large region encompassing *3′CBE-hs3b-hs4* as a whole, while our study based on mutant models allows to consider the *3′RR* and *3′CBE* as distinct functional entities acting as transcriptional enhancers and chromatin insulators, respectively. By also interrogating a poorly delineated yet useful model (*Δhs567* model), our study indeed demonstrates that *IgH* big loop interaction does not require the *3′CBE* region since such loops were efficiently formed in the absence of the *hs5-6-7* insulators (Supplementary Figures S7). Our results strongly suggests that the *IgH* TAD border is dispensable for loops preparing for CSR; this was in agreement with both studies describing the Δ*hs567* (36) and the Δ*3′CBEs* mouse models (5) (Supplementary Figure S7). A reasonable interpretation is that the *3′CBE* region acts as a ‘stop’ to prevent loop extrusion beyond the TAD boundary rather than as a loop-forming enhancer. While our study strongly supports the idea that *IgH* loops require the *3′RR*, it is reasonable to imagine that the proximity of the *3′RR* to the *3′CBE* (TAD boundary) prevents further extrusion and consequently locks the regulatory region at the loop terminus.

In extension to the loop extrusion-mediated CSR model proposed by the Alt’s group (18,19), our current study strongly suggests that core enhancers of the *3′RR* are majors players in cohesin loading. Whether this distal regulatory region and/or its main interactor (Eμ/Iμ region) recruit sequentially the putative NIPBL cohesin loader and the WAPL releaser remains an open question.

## Supplementary Material

gkae867_Supplemental_File

## Data Availability

Raw data from ATAC-seq, 3C-HTGTS and LAM-HTGTS have been deposited in the European Nucleotide Archive database under access number PRJEB59246.

## Notes

Present address: Morgane Thomas, Laboratoire Suivi des Thérapies Innovantes, Institut de Génétique Humaine, UMR 9002 CNRS-UM, Montpellier, France.
