# Supplementary material for: Core enhancers of the 3′RR optimize IgH nuclear position and loop conformation for successful oriented class switch recombination
Source: Nucleic Acids Res. 2024 Oct 16;52(20):12281–94. doi: 10.1093/nar/gkae867 (PMC11551739; doi:10.1093/nar/gkae867)
Supplement: gkae867_Supplemental_File [file gkae867_supplemental_file.pdf]

Figure S1

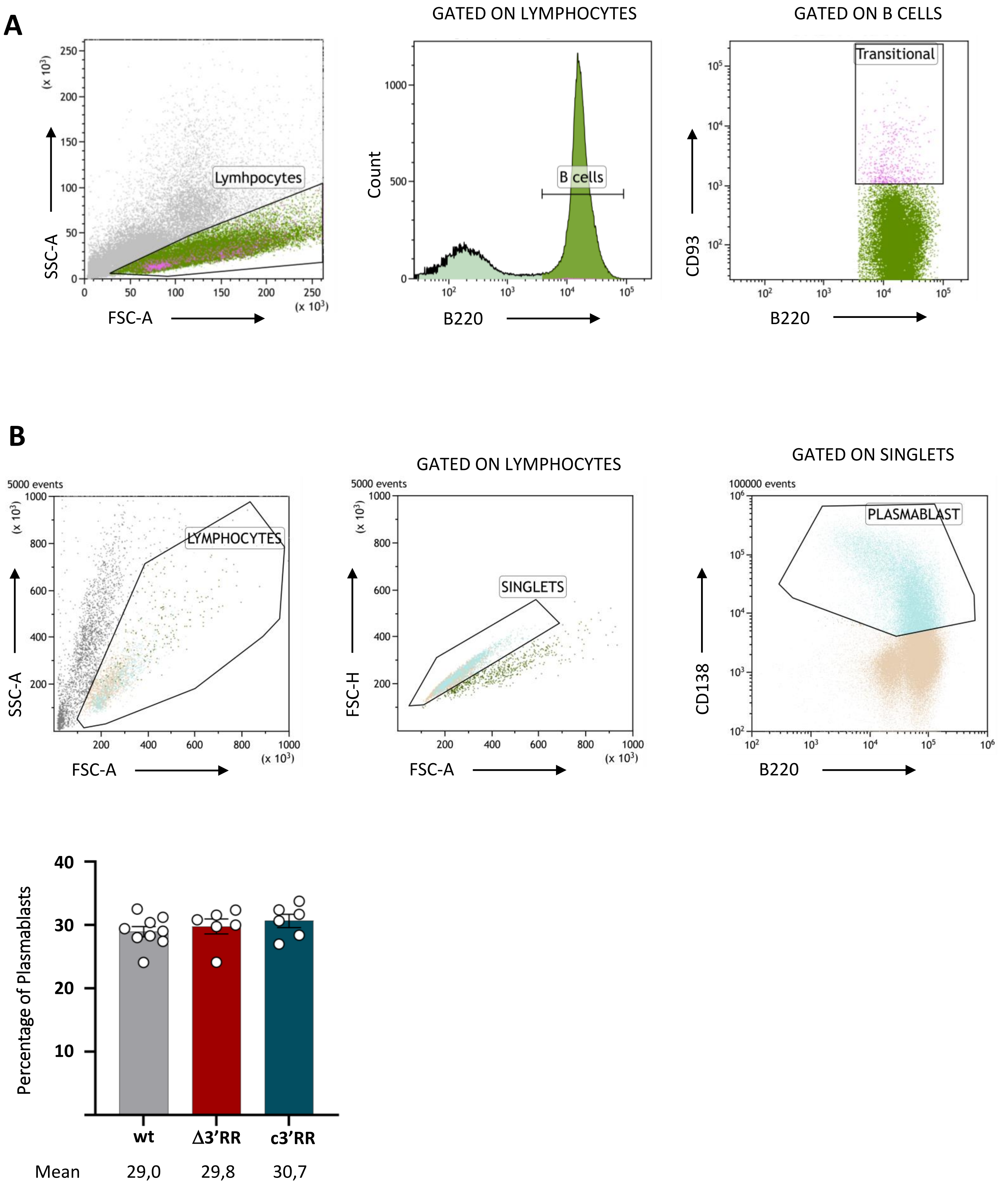
A
GATED ON LYMPHOCYTES
GATED ON B CELLS
Lymhpocytes
SSC-A
FSC-A
(x 10³)
B cells
Count
B220
Transitional
CD93
B
5000 events
LYMPHOCYTES
GATED ON LYMPHOCYTES
SINGLETS
FSC-H
GATED ON SINGLETS
100000 events
PLASMABLAST
CD138
Percentage of Plasmablasts
wt
Δ3'RR
c3'RR
Mean 29,0 29,8 30,7

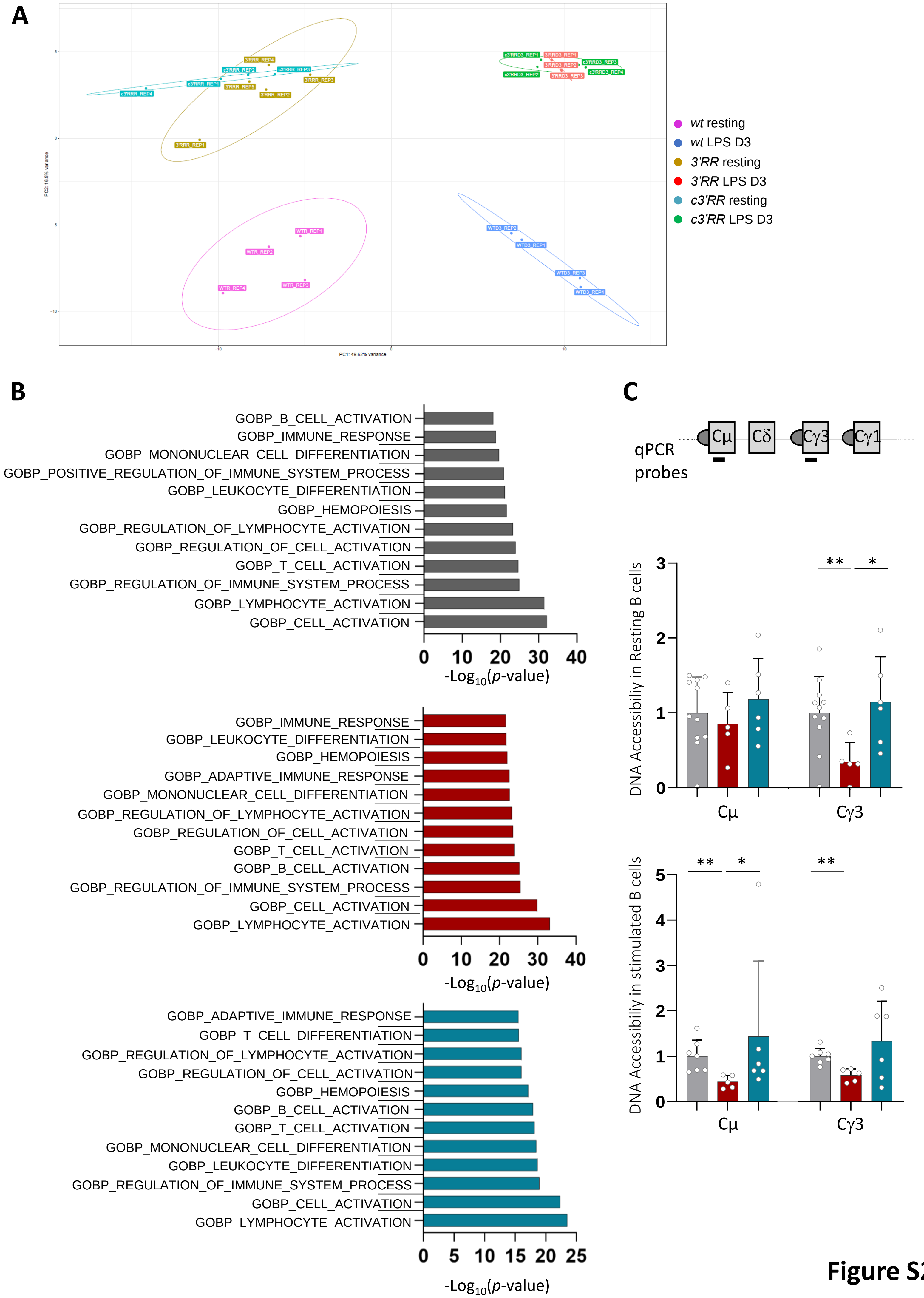
A
wt resting
wt LPS D3
3'RR resting
3'RR LPS D3
c3'RR resting
c3'RR LPS D3
B
GOBP_B_CELL_ACTIVATION
GOBP_IMMUNE_RESPONSE
GOBP_MONONUCLEAR_CELL_DIFFERENTIATION
GOBP_POSITIVE_REGULATION_OF_IMMUNE_SYSTEM_PROCESS
GOBP_LEUKOCYTE_DIFFERENTIATION
GOBP_HEMOPOIESIS
GOBP_REGULATION_OF_LYMPHOCYTE_ACTIVATION
GOBP_REGULATION_OF_CELL_ACTIVATION
GOBP_T_CELL_ACTIVATION
GOBP_REGULATION_OF_IMMUNE_SYSTEM_PROCESS
GOBP_LYMPHOCYTE_ACTIVATION
GOBP_CELL_ACTIVATION
-Log10(p-value)
GOBP_IMMUNE_RESPONSE
GOBP_LEUKOCYTE_DIFFERENTIATION
GOBP_HEMOPOIESIS
GOBP_ADAPTIVE_IMMUNE_RESPONSE
GOBP_MONONUCLEAR_CELL_DIFFERENTIATION
GOBP_REGULATION_OF_LYMPHOCYTE_ACTIVATION
GOBP_REGULATION_OF_CELL_ACTIVATION
GOBP_T_CELL_ACTIVATION
GOBP_B_CELL_ACTIVATION
GOBP_REGULATION_OF_IMMUNE_SYSTEM_PROCESS
GOBP_CELL_ACTIVATION
GOBP_LYMPHOCYTE_ACTIVATION
-Log10(p-value)
GOBP_ADAPTIVE_IMMUNE_RESPONSE
GOBP_T_CELL_DIFFERENTIATION
GOBP_REGULATION_OF_LYMPHOCYTE_ACTIVATION
GOBP_REGULATION_OF_CELL_ACTIVATION
GOBP_HEMOPOIESIS
GOBP_B_CELL_ACTIVATION
GOBP_T_CELL_ACTIVATION
GOBP_MONONUCLEAR_CELL_DIFFERENTIATION
GOBP_LEUKOCYTE_DIFFERENTIATION
GOBP_REGULATION_OF_IMMUNE_SYSTEM_PROCESS
GOBP_CELL_ACTIVATION
GOBP_LYMPHOCYTE_ACTIVATION
-Log10(p-value)
C
qPCR probes
Cμ
Cδ
Cγ3
Cγ1
DNA Accessibiliy in Resting B cells
DNA Accessibiliy in stimulated B cells
Cμ
Cγ3

Figure S2

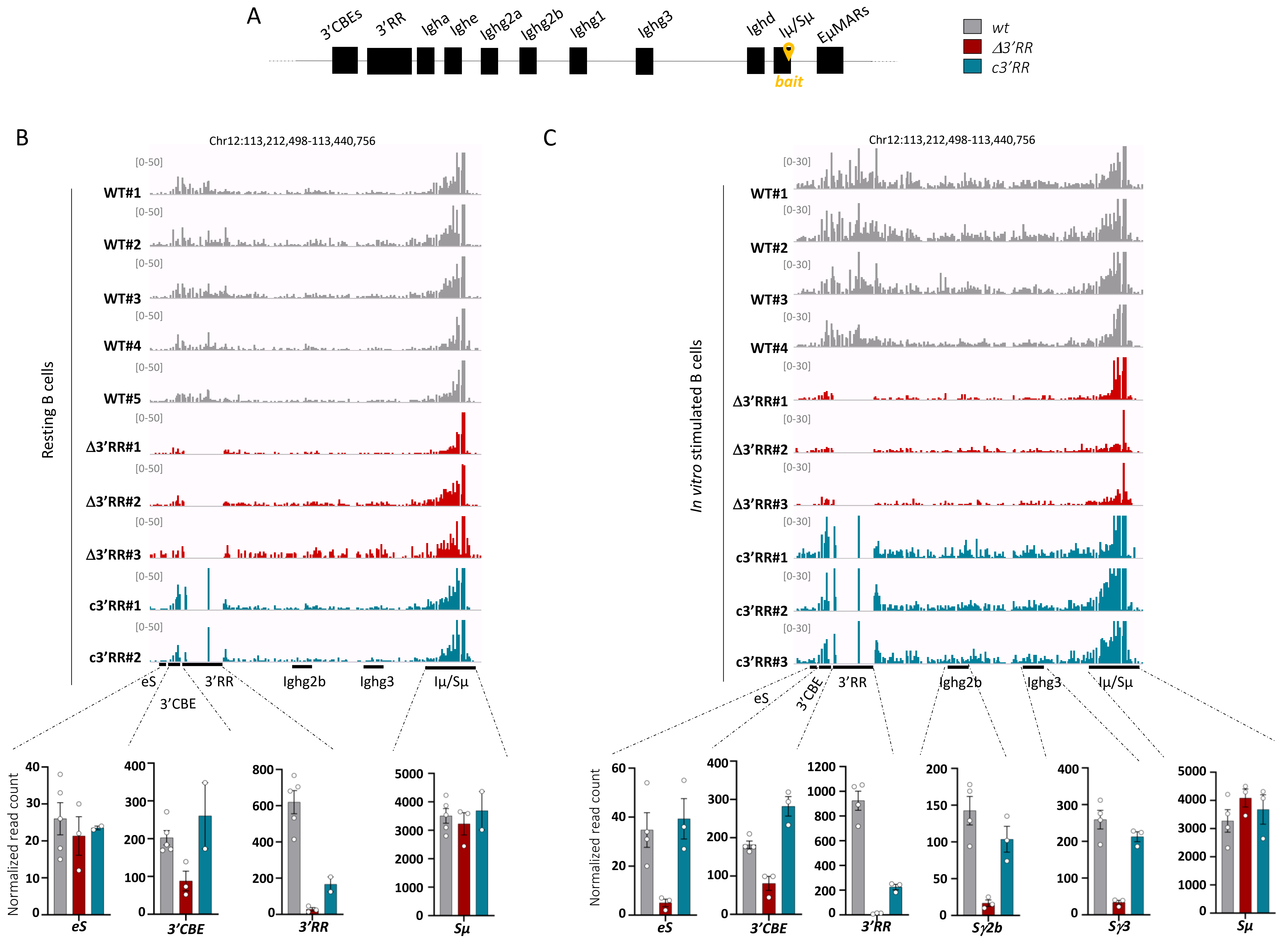
A
3'CBEs
3'RR
Igha
Ighe
Ighg2a
Ighg2b
Ighg1
Ighg3
Ighd
Iμ/Sμ
EμMARs
bait
wt
Δ3'RR
c3'RR
B
Chr12:113,212,498-113,440,756
Resting B cells
WT#1
WT#2
WT#3
WT#4
WT#5
Δ3'RR#1
Δ3'RR#2
Δ3'RR#3
c3'RR#1
c3'RR#2
[0-50]
eS
3'CBE
3'RR
Ighg2b
Ighg3
Iμ/Sμ
Normalized read count
eS
3'CBE
3'RR
Sμ
C
Chr12:113,212,498-113,440,756
In vitro stimulated B cells
[0-30]
WT#1
WT#2
WT#3
WT#4
Δ3'RR#1
Δ3'RR#2
Δ3'RR#3
c3'RR#1
c3'RR#2
c3'RR#3
eS
3'CBE
3'RR
Ighg2b
Ighg3
Iμ/Sμ
Normalized read count
eS
3'CBE
3'RR
Sγ2b
Sγ3
Sμ

Figure S3

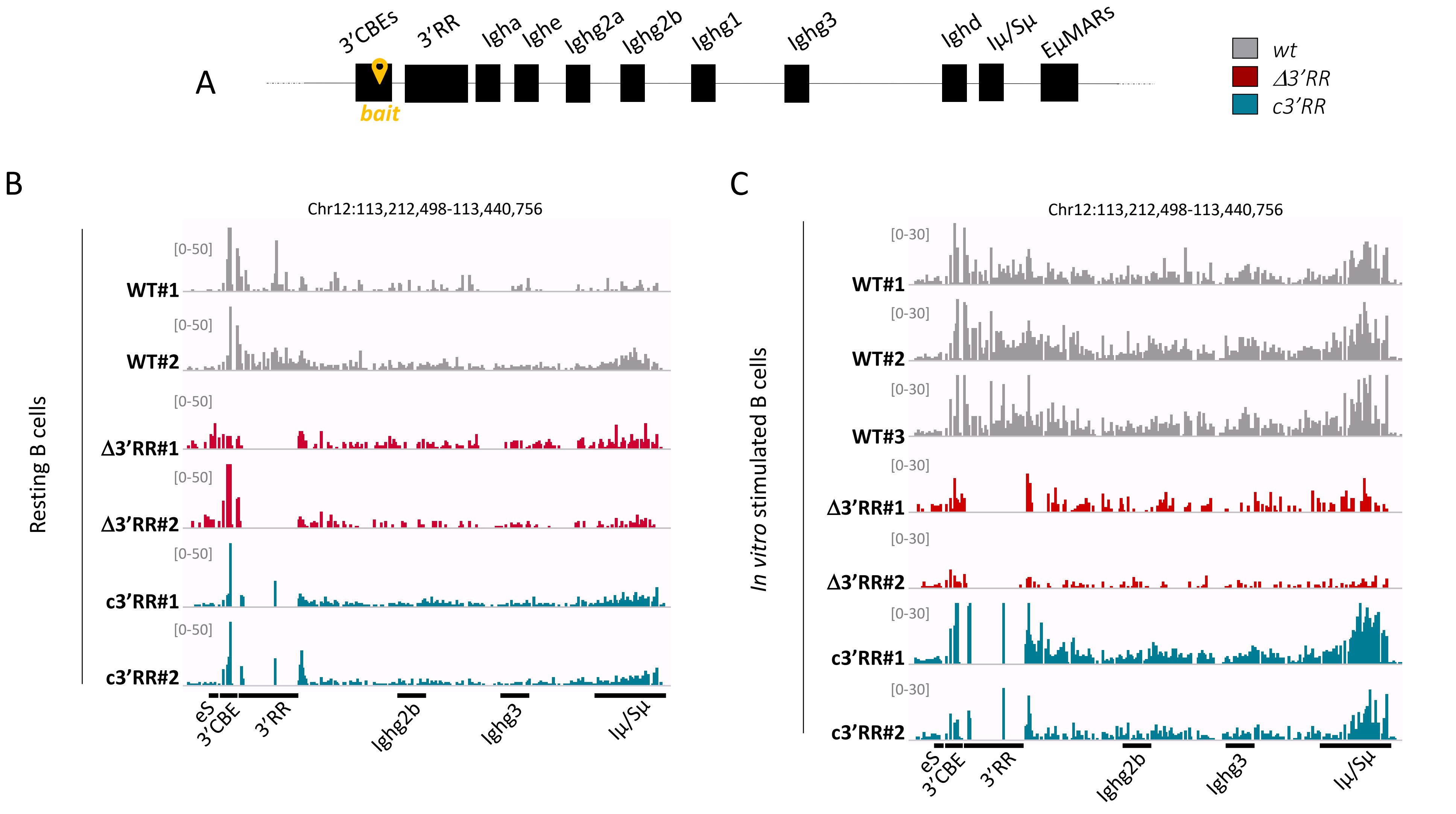

A
3'CBEs
3'RR
Igha
Ighe
Ighg2a
Ighg2b
Ighg1
Ighg3
Ighd
Iμ/Sμ
EμMARs
bait
wt
Δ3'RR
c3'RR
B
Chr12:113,212,498-113,440,756
Resting B cells
[0-50]
WT#1
WT#2
Δ3'RR#1
Δ3'RR#2
c3'RR#1
c3'RR#2
eS
3'CBE
3'RR
Ighg2b
Ighg3
Iμ/Sμ
C
Chr12:113,212,498-113,440,756
In vitro stimulated B cells
[0-30]
WT#1
WT#2
WT#3
Δ3'RR#1
Δ3'RR#2
c3'RR#1
c3'RR#2
eS
3'CBE
3'RR
Ighg2b
Ighg3
Iμ/Sμ

Figure S4

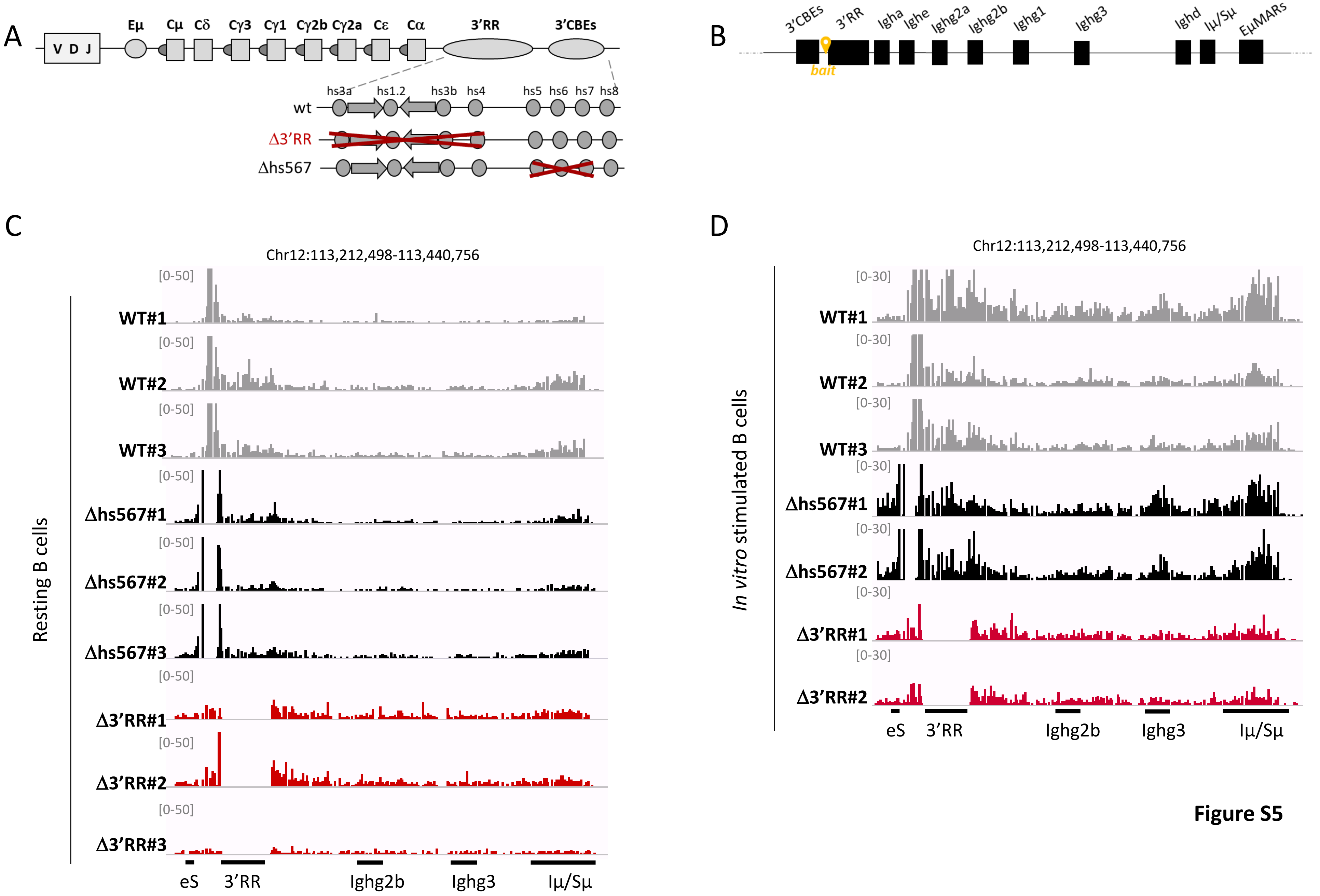

A
V D J
Eμ
Cμ
Cδ
Cγ3
Cγ1
Cγ2b
Cγ2a
Cε
Cα
3'RR
3'CBEs
hs3a
hs1.2
hs3b
hs4
hs5
hs6
hs7
hs8
wt
Δ3'RR
Δhs567
B
3'CBEs
3'RR
Igha
Ighe
Ighg2a
Ighg2b
Ighg1
Ighg3
Ighd
Iμ/Sμ
EμMARs
bait
C
Chr12:113,212,498-113,440,756
Resting B cells
[0-50]
WT#1
WT#2
WT#3
Δhs567#1
Δhs567#2
Δhs567#3
Δ3'RR#1
Δ3'RR#2
Δ3'RR#3
eS
3'RR
Ighg2b
Ighg3
Iμ/Sμ
D
Chr12:113,212,498-113,440,756
In vitro stimulated B cells
[0-30]
WT#1
WT#2
WT#3
Δhs567#1
Δhs567#2
Δ3'RR#1
Δ3'RR#2
eS
3'RR
Ighg2b
Ighg3
Iμ/Sμ

Figure S5

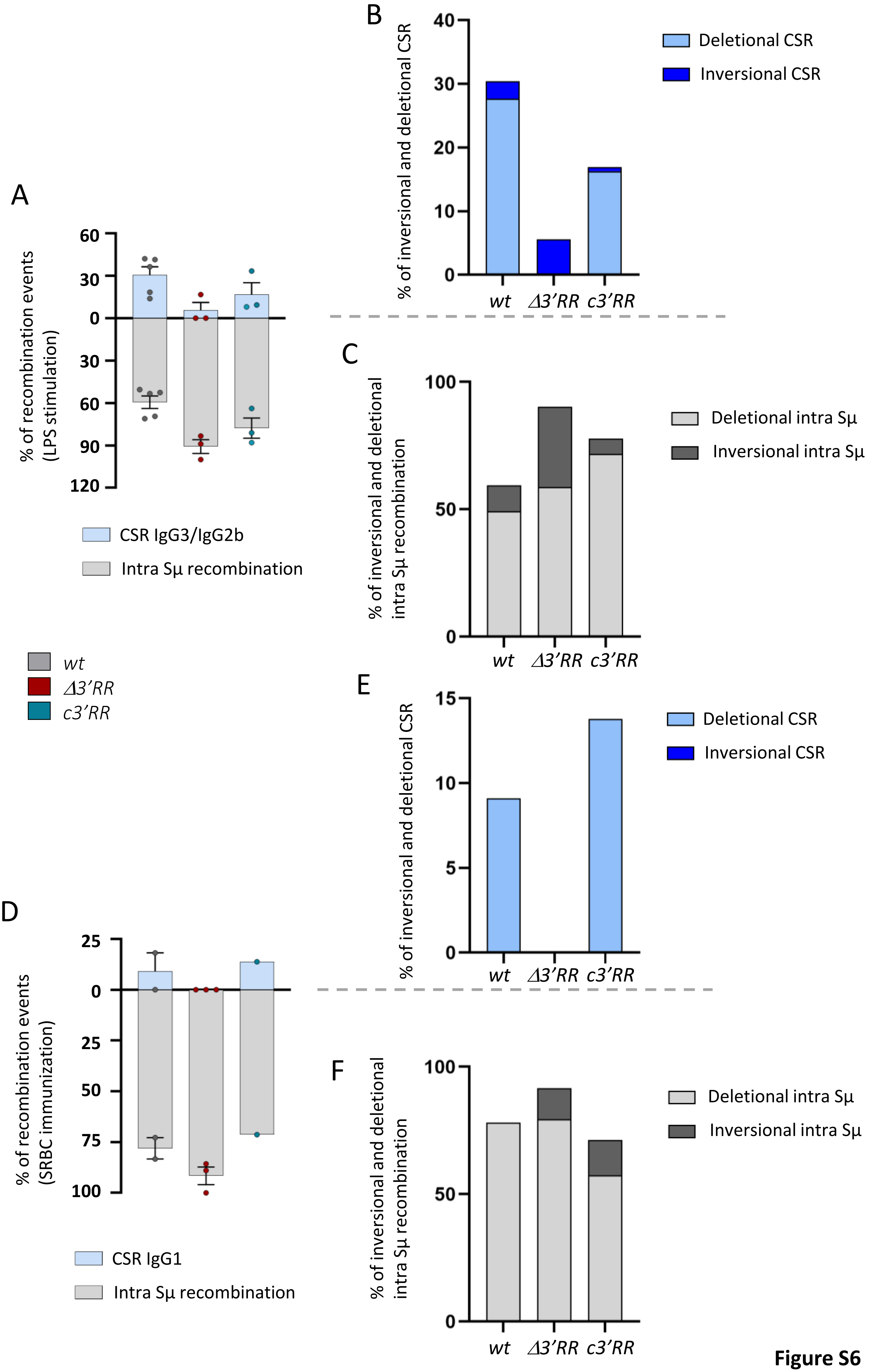
A
60
30
0
30
60
90
120
% of recombination events
(LPS stimulation)
CSR IgG3/IgG2b
Intra Sμ recombination
wt
Δ3'RR
c3'RR
B
% of inversional and deletional CSR
40
30
20
10
0
wt
Δ3'RR
c3'RR
Deletional CSR
Inversional CSR
C
% of inversional and deletional
intra Sμ recombination
100
50
0
wt
Δ3'RR
c3'RR
Deletional intra Sμ
Inversional intra Sμ
D
25
0
25
50
75
100
% of recombination events
(SRBC immunization)
CSR IgG1
Intra Sμ recombination
E
% of inversional and deletional CSR
15
10
5
0
wt
Δ3'RR
c3'RR
Deletional CSR
Inversional CSR
F
% of inversional and deletional
intra Sμ recombination
100
50
0
wt
Δ3'RR
c3'RR
Deletional intra Sμ
Inversional intra Sμ

Figure S6

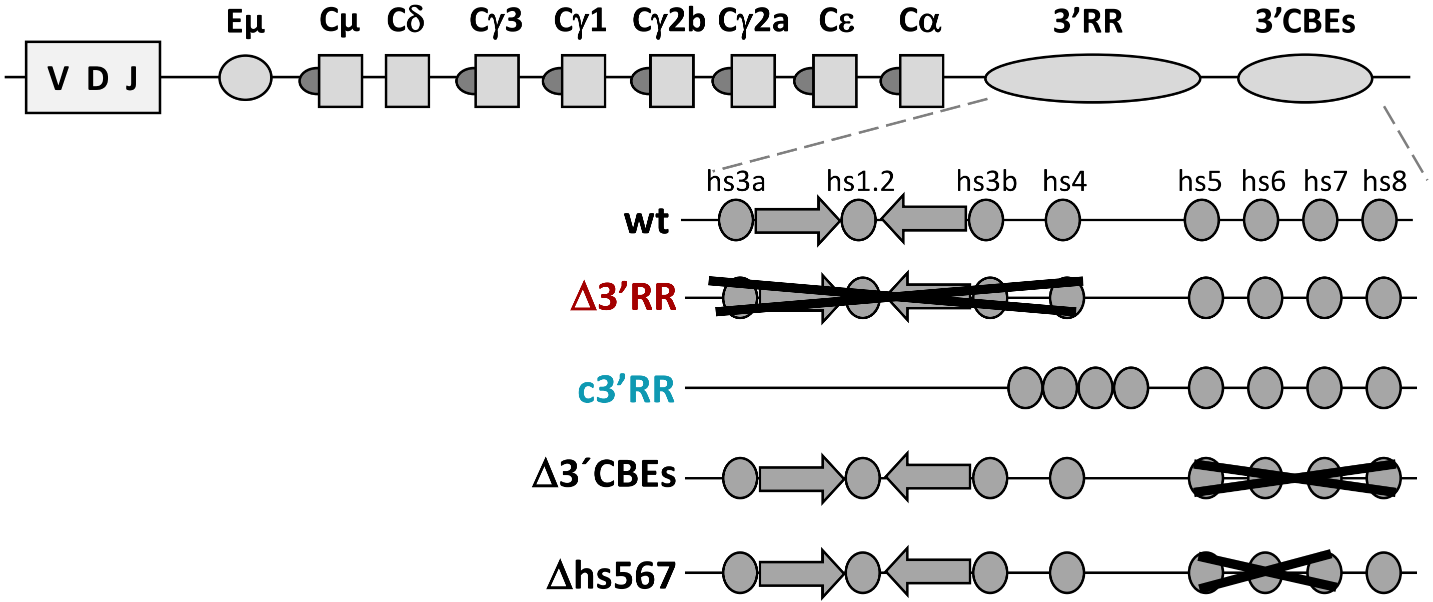
V D J
Eμ
Cμ
Cδ
Cγ3
Cγ1
Cγ2b
Cγ2a
Cε
Cα
3’RR
3’CBEs
hs3a
hs1.2
hs3b
hs4
hs5
hs6
hs7
hs8
wt
Δ3’RR
c3’RR
Δ3´CBEs
Δhs567

Figure S7

Supplementary Figure S1: **A**. Gating strategy to sort transitional B cells from spleen. **B**. *Top*: gating strategy to identify plasmablasts in *in vitro* stimulated splenic B cells. *Bottom*: bargraphs represent the percentage of plasmablasts in *wt*, *Δ3'RR* and *c3'RR* mice (n=6-9).

Supplementary Figure S2: A. Principal Component Analysis of ATAC-seq of resting B cells (R, n=4-5) and *in vitro* LPS stimulated B cells for three days (D3, n=3-4) from *wt*, *Δ3'RR* and *c3'RR* mice. B. Top twelve of enriched pathways from ChIP-Enrich based on DO regions. **C**. *Top*: qPCR probes are represented on *IgH* locus (black lines). Relative DNA accessibility of *Cμ* and *Cγ3* regions in resting (middle) and stimulated B cells (bottom) measured by qPCR assays on transposed DNA libraries. Normalization was done with relatively to *Slc19a2* which is not differentially opened between models. Each point represents an independent biological replicate (resting B cells: *wt* n=11, *Δ3'RR* n=5, *c3'RR* n=6 mice and stimulated B cells*: wt* n=17 *Δ3'RR* n=5, *c3'RR n=6 mice*). Error bars represent SD, *p*-value was determined by a two-tailed Mann-Whitney test; only significant differences are indicated *p<0,05, **p<0,01.

Supplementary Figure S3: A. Scheme of the murine *IgH* locus, location of the *Iμ/Sμ* bait used for 3C-HTGTS is indicated by a yellow location pin. **B**. *Top*: Bedgraphs, visualized by IGV tool, showing chromatin interactions in replicates of splenic resting B cells from independent *wt* (n=5)*, Δ3'RR* (n=3) and *c3'RR* (n=2) mice. *Bottom*: Bargraphs displaying the normalized read count in specific regions outside and inside the *IgH* locus: *eS* (outside *IgH*), *3'RR, 3'CBE* and *Sμ*. **C**. *Top*: Same as in (**B**) for *in vitro* stimulated B cells from independent *wt* (n=4), *Δ3'RR* (n=3) and *c'3RR* (n=3) mice. Scale was adjusted to show interactions of interest. *Bottom*: same as in (**B**), includes also counts in *IgH I-Cγ2b* and *I-Cγ3* regions subject to LPS-dependent regulation.

Supplementary Figure S4: **A**. Scheme of the murine *IgH* locus, location of the *3'CBE* bait used for 3C-HTGTS is shown by a yellow location pin. **B**. Bedgraphs, visualized by IGV tool, showing chromatin interactions in replicates of splenic resting B cells from independent *wt* (n=2)*, Δ3'RR* (n=2) and *c3'RR* (n=2) mice. **C**. Same as in (**B)** for *in vitro* stimulated B cells from mice of *wt* (n=3), *Δ3'RR* (n=2) and *c'3RR* (n=2). Scale was adjusted to show interactions of interest.

Supplementary Figure S5: **A**. Scheme of murine *IgH* locus including the components of the *3'RR* and the *3'CBE* regions. Deletions for each model (*3'RR* (1, 2) *and hs567* (3)) are represented by a red cross **B**. Scheme of the murine *IgH* locus, location of the *3'RR* bait used for 3C-HTGTS is shown by a yellow location pin. **C**. Bedgraphs, visualized with IGV tool, showing chromatin interactions in replicates of splenic resting B cells from independent *wt* (n=3)*, Δ3'RR* (n=3) and *Δhs567* (n=3) mice. **D**. Same as in **C** for *in vitro* stimulated B cells from mice of *wt* (n=3), *Δ3'RR* (n=2) and *Δhs567* (n=2) models. Scale was adjusted to show interactions of interest.

Supplementary Figure S6: A. Bargraphs representing the proportion of CSR (blue) and Intra-Sμ (grey) recombination in *in vitro* stimulated splenic B cells from independent *wt* (n=5), *Δ3'RR* (n=3) and *c3'RR* (n=3) mice. Each individual mouse is represented by a colored dot. **B**. Bargraphs displaying the proportion of deletional (light blue) and inversional CSR (dark blue) in *in vitro* stimulated splenic B cells from independent *wt* (n=5), *Δ3'RR* (n=3) and *c3'RR* (n=3) mice. **C**. Bargraphs showing the proportion of deletional (light grey) and inversional (dark grey) intra-Sμ recombination events in *in vitro* stimulated splenic B cells from independent *wt* (n=5), *Δ3'RR* (n=3) and *c3'RR* (n=3) mice. **D**. Same as in (**A**) for SRBC-immunized *wt* (n=2), *Δ3'RR* (n=3) and *c3'RR* (n=1) mice. **E**. Same as in (**B**) for SRBC-immunized *wt* (n=2), *Δ3'RR* (n=3) and *c3'RR* (n=1) mice. **F**. Same as in (**C**) for SRBC-immunized *wt* (n=2), *Δ3'RR* (n=3) and *c3'RR* (n=1) mice.

Supplementary Figure S7: Scheme of the murine *IgH* locus including the *3'RR* and *3'CBE* components. Deletions for each model used in this study (*Δ3'RR* (1, 2), *c'3RR* (4) *and Δhs567* (3)) or cited in the discussion (*Δ3'CBE* (5)). are represented by a black cross

Table S1 : Number of differentially opened regions by ATAC-Seq

A

	Number of Differentially Opened regions
WT R *vs* WT S	4899
c3'RR R *vs* c3'RR S	9617
3'RR R *vs* 3'RR S	4797

B

	Number of Differentially Opened regions	
	**Resting**	***In vitro* stimulated**
WT *vs* c3'RR	323	331
WT *vs* Δ3'RR	124	108
c3'RR *vs* Δ3'RR	187	174

Table S2 : Coordinates used for quantification of normalized coverage in R1, R2 and R3 regions

	chr12	
**R1 - Sµ**	113 407 000	113 430 000
**R2 - Constant**	113 255 000	113 407 000
**R3 - 3'CBE**	113 215 000	113 225 000

Table S3 : Normalized reads count of 3C-HTGTS performed with *Iµ/Sµ* bait in region of interest within the *IgH* locus in resting and stimulated B cells from *wt* and mutants mice

A

	chr12	
**Sµ**	113 385 000	113 430 000
**Sγ3**	113 355 000	113 370 000
**Sγ2b**	113 315 000	113 300 000
**3'RR**	113 255 000	113 225 001
**3'CBE**	113 225 000	113 215 001
**eS**	113 215 000	113 209 000
**Whole IgH**	113 209 000	133 430 000

B

	**Resting B cells**									
	**WT#1**	**WT#2**	**WT#3**	**WT#4**	**WT#5**	**Δ3RR#1**	**Δ3RR#2**	**Δ3RR#3**	**c3'RR#1**	**c3'RR#2**
**Sµ**	2802	3538	3867	3096	4233	3588	2445	3651	3015	4365
**3'RR**	767	704	532	681	414	46	24	14	207	125
**3'CBE**	271	216	169	184	172	52	139	73	348	173
**eS**	33	15	26	38	18	22	30	12	23	24
**Whole IgH**	5066	5157	5176	5401	5359	4561	3522	4035	4479	5315

C

	***In vitro* stimulated B cells**									
	**WT#1**	**WT#2**	**WT#3**	**WT#4**	**Δ3RR#1**	**Δ3RR#2**	**Δ3RR#3**	**c3'RR#1**	**c3'RR#2**	**c3'RR#3**
**Sµ**	4214	3527	3043	2313	3459	4299	4503	4315	4076	2643
**Sγ3**	191	258	169	276	39	49	22	228	224	186
**Sγ2b**	94	129	178	169	25	15	10	135	102	74
**3'RR**	717	891	1037	1051	14	6	15	251	244	182
**3'CBE**	164	176	210	177	99	44	99	233	294	319
**eS**	20	34	31	54	7	2	6	27	36	55
**Whole IgH**	6057	5941	5987	5177	3870	4508	4771	6252	5951	4168

Table S4 : Coordinates used for LAM jonctions analysis

	chr12	
**Sμ**	113 418 000	113 426 676
**Sγ3**	113 357 000	113 366 000
**Sγ1**	113 325 000	113 338 000
**Sγ2b**	113 304 000	113 314 000
